# Life-course socioeconomic position and health in older adulthood: a causal mediation analysis in the 1958 British Birth Cohort

**DOI:** 10.1016/j.lanepe.2026.101890

**Published:** 2026-10-15

**Authors:** Yiling Guo, Alina Pelikh, George B. Ploubidis, Alissa Goodman

**Affiliations:** Centre for Longitudinal Studies, Social Research Institute, University College London, United Kingdom

**Keywords:** Socioeconomic position, Health inequalities, Life-course epidemiology, Ageing, Causal mediation analysis, Birth cohort study

## Abstract

**Background:**

Childhood socioeconomic position (SEP) influences later health, but how inequalities persist into early older age and operate through adult SEP remains unclear. Quantifying these pathways is important for identifying opportunities to reduce health inequalities over the life course. We therefore used an interventional causal mediation framework to quantify the contribution of four distinct dimensions of adult SEP to childhood socioeconomic inequalities in self-reported and objective health outcomes in early older age.

**Methods:**

We analysed data from the 1958 British Birth Cohort collected at birth, ages 33 and 55, and the most recent sweep, when participants were aged 60–66 years. Sample sizes ranged from 4014 to 8363 depending on the outcome. We examined the extent to which four dimensions of adult SEP — adult occupational class, education, housing tenure, and income — mediated associations between childhood SEP, measured by paternal occupational class at birth, and self-rated health (SRH), C-reactive protein (CRP), cholesterol ratio, glycated haemoglobin (HbA1c), and NT-proBNP.

**Findings:**

Among men, occupational class mediated 39% of the association with poorer SRH (indirect effect RR 0.90, 95%: CI 0.86–0.95), and education mediated 27% (0.93, 0.90–0.96). Among women, mediation was smaller and most evident via education (10%; 0.95, 0.91–0.98) and housing tenure (6%; 0.97, 0.94–0.99). Mediation was limited for CRP and minimal for cholesterol ratio, HbA1c, and NT-proBNP.

**Interpretation:**

Improving adult SEP could reduce, but is unlikely to eliminate, early older-age health inequalities associated with childhood SEP, highlighting the importance of interventions across the life course.

**Funding:**

UK Economic and Social Research Council.

**Research in context**

**Evidence before this study**

We searched PubMed and Google Scholar from database inception to June 10, 2026, for studies examining childhood or early-life socioeconomic position (SEP), adult SEP, and later-life health, with a particular focus on prospective cohort studies and studies assessing mediation by adult SEP. We used combinations of the terms “childhood SEP”, “early-life SEP”, “adult SEP”, “socioeconomic position”, “mediation”, “life course”, “ageing”, “health outcomes”, “self-rated health”, and “biomarkers.” No language restrictions were applied, and we screened the reference lists of relevant articles and reviews. We identified a substantial body of prospective evidence, particularly from British birth cohort studies, demonstrating that childhood socioeconomic disadvantage is associated with poorer health across adulthood and into early older age. Previous studies consistently found that adult SEP partly explains these associations, but most relied on attenuation following mutual adjustment or conventional path-based mediation methods. Only one previous study formally quantified mediation using data from the 1958 National Child Development Study (NCDS), focusing on frailty in midlife and a single latent measure of adult SEP. We found no prospective study that used an interventional mediation framework to quantify the contribution of distinct dimensions of adult SEP to childhood socioeconomic inequalities across multiple health outcomes in early older age.

**Added value of this study**

Using more than six decades of prospective data from the NCDS, we applied an interventional mediation framework to quantify the extent to which childhood socioeconomic inequalities in health in early older age operate through four distinct dimensions of adult SEP: occupational class, education, housing tenure, and income. Unlike conventional mediation approaches, the interventional framework accommodates exposure-induced mediator–outcome confounding and exposure–mediator interaction, providing policy-relevant estimates of the direct and indirect contributions of different socioeconomic pathways. The study extends previous prospective evidence from midlife into early older age and examines a broad range of outcomes, including self-rated health (SRH) and objective biomarkers. We found that mediation through adult SEP was evident for SRH, modest for C-reactive protein, and minimal for cholesterol ratio, HbA1c, and NT-proBNP. These findings extend existing evidence by showing how the estimated contribution of measured adult socioeconomic circumstances varies across health outcomes in early older age. Quantifying these pathways in early older age is particularly relevant because health inequalities at this stage have important implications for work capacity, economic security, and healthy ageing around the transition to retirement.

**Implications of all the available evidence**

Together with previous evidence, our findings suggest that childhood socioeconomic disadvantage continues to influence health into early older age, with adult socioeconomic circumstances explaining only a small part of these inequalities. The persistence of substantial direct effects indicates that interventions targeting adult socioeconomic disadvantage are likely to reduce, but not eliminate, inequalities originating in childhood, highlighting the importance of policies acting across the life course.

## Introduction

Population ageing presents a major challenge for health systems, pension and social policy. Health in older age is shaped by processes that begin early in life and unfold across the life course,^1^^,^^2^ making it essential to understand the early-life determinants of healthy ageing, the years lived in good health. British birth cohort evidence shows a strong childhood SEP gradient in premature mortality: those from disadvantaged social class origins face roughly 1.7–2.7 times the risk of death in adulthood and early old age compared with those from advantaged origins.^3^ Similar gradients have been reported elsewhere, including the U.S. and Northern Europe.^4^

Childhood SEP may shape later health through multiple interconnected pathways, including material conditions, health behaviours, psychosocial processes, and biological embedding. Life-course epidemiology proposes several complementary models to explain these associations. The critical-period model proposes that exposure during a specific developmental window has lasting effects on health, whereas the same exposure outside that window does not confer the same excess risk.^5^ Accumulation and pathway hypotheses emphasise the cumulative and sequential influences of socioeconomic circumstances across the life course, with adult SEP representing one important modifiable pathway linking childhood (dis)advantage to health in later life.^5^

These questions have been examined extensively in both international and UK studies.^6^, ^7^, ^8^, ^9^, ^10^, ^11^, ^12^ British birth cohorts have made particularly important contributions because of their prospective follow-up from birth and repeated measurement of socioeconomic circumstances and health across multiple life stages. Collectively, these studies show that childhood socioeconomic disadvantage is associated with poorer health from midlife into early older age and that adult SEP explains part, but not all, of these associations.^7^, ^8^, ^9^, ^10^, ^11^, ^12^ Additional studies are summarised in Appendix Table A1.

Despite this substantial evidence, important methodological gaps remain. Most previous studies have relied on attenuation following mutual adjustment for childhood and adult SEP,^6^^,^^7^^,^^10^^,^^11^ while fewer have applied conventional product-of-coefficients path mediation methods.^8^^,^^9^ Although these approaches have provided important evidence supporting the concurrent operation of critical-period, accumulation and pathway processes, they do not formally quantify the direct and indirect components of the association and rely on assumptions difficult to satisfy in life-course settings, including no exposure–mediator and no exposure-induced mediator–outcome confounding.^13^ Rogers et al. (2021) advanced this literature by formally quantifying mediation using a latent measure of adult SEP in relation to midlife frailty.^12^ We extend this literature by applying an interventional mediation framework to examine four adult SEP indicators separately in relation to self-rated health and biomarkers in early older age.

Understanding these pathways in early older age is particularly important because biological embedding and accumulated health risks may make the consequences of childhood socioeconomic disadvantage increasingly apparent with age, potentially altering both the strength of childhood SEP associations and the extent to which they are mediated through adult socioeconomic circumstances.^14^^,^^15^ Health at this stage also has important implications for work capacity, economic security and healthy ageing around the transition to retirement.

To address these gaps, we used data from the 1958 National Child Development Study (NCDS) to quantify the extent to which childhood socioeconomic inequalities in health in early older age operate through adult SEP. Using an interventional causal mediation framework, we estimated interventional indirect effects operating through four distinct dimensions of adult SEP—occupational class, education, housing tenure, and income—as well as interventional direct effects representing the remaining association not operating through these pathways. By combining a formal mediation framework with multiple indicators of adult SEP and a broad range of health outcomes—including self-rated health and objective biomarkers—our study provides new evidence on the extent to which measured adult socioeconomic circumstances mediate childhood socioeconomic inequalities in health in early older age.

## Methods

### Data

The study used the 1958 National Child Development Study (NCDS),^16^ a nationally representative longitudinal study of 17,415 individuals born in Britain during one week in 1958. Data were drawn from birth, age 33, age 55, and the most recent sweep, when cohort members were aged 60–66 years. In this latest sweep, 8405 participants completed the main survey and 6309 provided blood samples.

### Outcome

We selected complementary subjective and objective measures of health to capture both overall health status and key physiological systems implicated in the biological embedding of socioeconomic disadvantage and subsequent morbidity. The blood-based biomarkers were chosen to capture processes central to healthy ageing: systemic inflammation (C-reactive protein [CRP]); lipid-related cardiovascular risk (cholesterol ratio); glycaemic regulation (glycated haemoglobin [HbA1c]); and cardiac strain (N-terminal pro–B-type natriuretic peptide [NT-proBNP]).^17^^,^^18^ Self-rated health (SRH) was included as a complementary global assessment of perceived physical and mental health and is an established predictor of morbidity and mortality independently of objective clinical measures.^19^

CRP was dichotomised as <3 mg/L (no inflammation = 0) versus 3–10 mg/L (inflammation = 1); values > 10 mg/L were excluded to minimise the influence of acute infection. The cholesterol ratio was calculated by dividing total cholesterol by high-density lipoprotein cholesterol. HbA1c was expressed as a percentage. NT-proBNP was measured in pg/mL and log-transformed. Add-back corrections were applied to cholesterol ratio and HbA1c values among participants taking relevant medications (Appendix Text A1). Higher cholesterol ratio, HbA1c, and NT-proBNP values indicated poorer health. SRH was dichotomised as good (excellent/very good/good = 0) versus fair/poor (=1).

### Life-course SEP

Childhood SEP was based on paternal occupational class at birth, classified according to the Registrar General’s Social Class (RGSC) scheme as non-manual (I, II, and IIIN) versus manual (IIIM, IV, and V). Adult SEP at age 55 was operationalised using occupational class (manual/non-manual), housing tenure (owner/renter), educational attainment (degree or higher/no degree), and income, allowing their relative contributions as mediators to be compared.

### Directed acyclic graph and confounders

Fig. 1 presents the directed acyclic graph (DAG) outlining the assumed causal relationships among confounders, childhood SEP, adult SEP, and health outcomes. Measures were timed to preserve temporal ordering and allow life-course pathways to unfold. Childhood SEP was the exposure and was hypothesised to influence later-life health directly and indirectly via adult SEP. Baseline confounders included ethnicity (Black and minority ethnic [BME]/non-BME) and grandparental social class (RGSC), reflecting demographic and intergenerational social advantage that precede and may confound SEP trajectories and later health. Exposure-induced mediator–outcome confounders measured at age 33 included smoking (yes/no), regular alcohol use (yes/no), low exercise (yes/no), psychological distress (standardised malaise score), long-standing illness (yes/no), marital status (married/not), and number of children, as these factors may be influenced by childhood SEP and affect both adult SEP and later health. Summary statistics are presented in Appendix Tables A2 and A3.

**Fig. 1:**
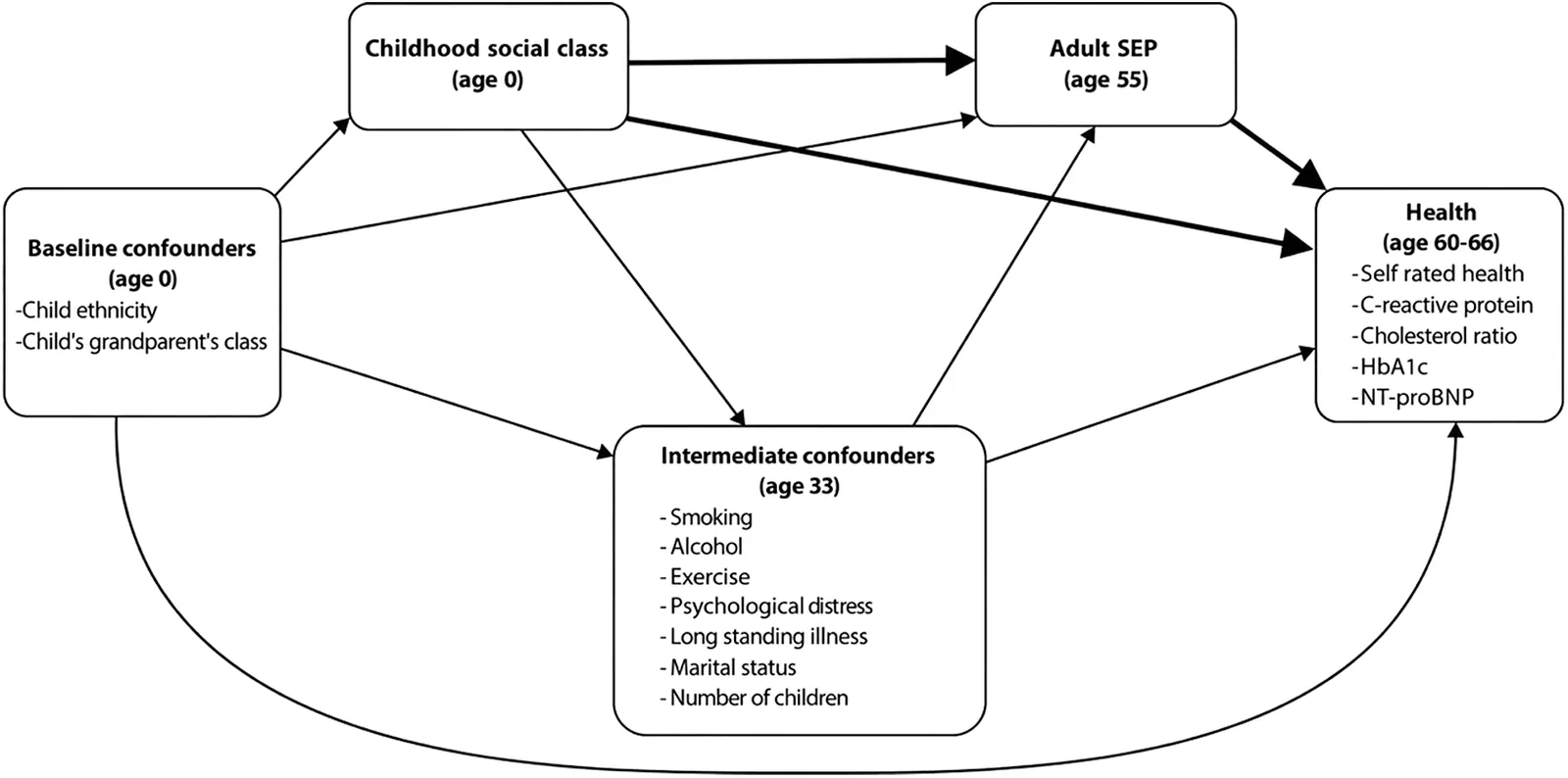
Directed acyclic graph (DAG) depicting the assumed causal structure, bold arrows indicate the estimands of interest.

Our primary estimands were the interventional indirect effect and direct effect. The interventional indirect effect captures the component operating via adult SEP. The interventional direct effect represents the remaining childhood SEP and health association not captured by adult SEP as measured and modelled here. Causal interpretation additionally assumes no unmeasured confounding conditional on variables included in Fig. 1, together with positivity, consistency, and no interference. These assumptions require sufficient variation in exposure and mediator values within confounder strata (positivity); that a participant’s observed outcome reflects the exposure and mediator values they actually experienced (consistency); and that one participant's exposure or mediator does not influence another participant's outcome (no interference).^20^ Further details on the estimands, identification assumptions, and estimation procedure are provided in Appendix Text A2.

### Statistical analysis

We first summarised the health outcomes overall and by childhood SEP, reporting proportions for binary outcomes and means for continuous outcomes, together with the manual minus non-manual difference. We then estimated associations between childhood SEP and each health outcome using regression models adjusted for baseline confounders. Mediation analyses were conducted separately by sex and for each adult SEP indicator, estimating interventional direct and indirect effects of childhood SEP on health while adjusting for all confounders. Outcome models included childhood SEP–adult SEP interactions, allowing the association between childhood SEP and later-life health to vary by adult SEP.

Interventional effects were estimated using a g-formula approach with direct counterfactual imputation. The interventional indirect effect compares outcomes when, within levels of baseline confounders, the distribution of each adult SEP mediator is shifted from that expected under disadvantaged childhood SEP to that expected under advantaged childhood SEP, while holding childhood SEP fixed. The interventional direct effect compares outcomes across childhood SEP while holding the mediator distribution fixed. Intermediate confounders are handled by averaging over their distribution conditional on childhood SEP and baseline confounders, allowing identification without the cross-world assumptions required for natural effects.^21^^,^^22^ The resulting estimands correspond to well-defined hypothetical interventions on the adult SEP distribution, enhancing policy relevance (Table 1). Mediation analyses were performed using the CMAverse R package with additional g-formula functionalities provided by CMAversePlus.^23^^,^^24^ Confidence intervals were obtained via bootstrapping.

**Table 1:** Interpreting patterns of interventional effects.

Pattern of effects	Interpretation	Implications
Small direct effect; large indirect effect	Most of the association operates via the mediator.	Improving adult SEP may substantially offset childhood disadvantage.
Large direct effect; small indirect effect	The association is unexplained by the mediator.	Intervening on adult SEP alone will be insufficient; early-life interventions are important.
Both direct and indirect effects are moderate	Multiple pathways contribute meaningfully.	A combined early-life and adult-life strategy may be required.
Both direct and indirect effects are null	No clear mediated or direct pathway.	Interventions on either SEP unlikely to change the outcome.

Like all longitudinal studies, the 1958 cohort has experienced loss to follow-up. For regression-based analyses, we addressed missing data among sweep respondents using multiple imputation by chained equations (MICE) and accounted for sweep non-response using inverse-probability weights (IPWs).^25^^,^^26^ For mediation analyses, missing data among sweep respondents were handled using Boot-MI, in which bootstrap resampling is followed by multiple imputation within each bootstrap sample.^27^ Because no established approach exists for combining IPWs with g-computation and Boot-MI, we assessed potential bias from sweep non-response by repeating the mediation analyses in a multiply imputed dataset comprising all cohort members who were alive and had not emigrated before the most recent sweep, including both respondents and non-respondents (n = 14,775). The results were materially unchanged (Appendix Tables A6 and A7). More information on the missing-data strategy is provided in the Appendix (Appendix Text A3, Appendix Tables A4 and A5).

Age at outcome assessment ranged from 60 to 66 years. Regression models therefore additionally adjusted for age at assessment. For mediation analyses, we assessed the influence of assessment timing in a restriction-based sensitivity analysis limited to participants assessed during 2022–2023 (Appendix Tables A13 and A14). Analyses included participants with non-missing outcome data; the derivation of the outcome-specific analytic samples is shown in Appendix Fig. A1. All analyses were conducted using Stata 18.5 and R.

### Ethics approval

This study is based on secondary analysis of anonymised NCDS data obtained through the UK Data Service End User License. As the analyses used existing de-identified data, no additional ethical approval was required. Ethical approval for the 1958 National Child Development Study was obtained from the relevant National Health Service Research Ethics Committees. All cohort members provided informed consent to participate in the study.

### Role of the funding source

The funders had no role in study design, data collection, data analysis, data interpretation, or writing of the report.

## Results

Table 2 presents descriptive statistics among participants with non-missing outcome data and observed childhood SEP. Compared with participants from advantaged childhood SEP, those from disadvantaged childhood SEP had a higher prevalence of poorer SRH and inflammation, a less favourable cholesterol ratio, and higher HbA1c. Little difference was observed for log-transformed NT-proBNP. Table 3 presents regression estimates for the association between childhood SEP and later-life health. Adjustment for baseline confounders and age attenuated some associations, although several remained evident. Among men, advantaged childhood SEP was associated with lower odds of poorer SRH and elevated CRP; associations with cholesterol ratio and HbA1c were in the same favourable direction, but confidence intervals included the null. The cholesterol–ratio association was clearer among women than men. Among women, associations were generally stronger, with advantaged childhood SEP associated with lower odds of poorer SRH and elevated CRP, and with lower cholesterol ratio.

**Table 2:** Health outcomes by childhood SEP.

Outcome	Overall	Non-manual	Manual	SEP difference (CI)
Poor/fair self-rated health, n (%)	1933 (25.6)	463 (19.3)	1470 (28.6)	9.3 (7.3, 11.3)
Presence of inflammation, n (%)	803 (22.1)	193 (16.1)	610 (25.1)	9.0 (6.3, 11.7)
Cholesterol ratio, mean (SD)	4.03 (1.29)	3.88 (1.22)	4.11 (1.32)	0.23 (0.15, 0.31)
Glycated haemoglobin (HbA1c) (%), mean (SD)	6.05 (1.05)	5.93 (0.82)	6.11 (1.15)	0.17 (0.11, 0.23)
ln [NT-proBNP (pg/mL)], mean (SD)	4.15 (0.89)	4.16 (0.88)	4.15 (0.90)	−0.01 (−0.07, 0.04)

Notes: Data are n (%) or mean (SD). NT-proBNP values were natural-log transformed. SEP differences (manual minus non-manual social class) are expressed as absolute prevalence differences (percentage points) for binary outcomes and mean differences for continuous outcomes. 95% CIs were estimated from unadjusted linear regression models with robust standard errors.

**Table 3:** Regression-based estimates of associations between childhood SEP (non-manual versus manual social class [ref]) and health.

Panel A. Men					
	Self-rated health OR (CI) (n = 4183)	C-reactive protein OR (CI) (n = 1996)	Cholesterol ratio β (CI) (n = 2479)	Glycated haemoglobin β (CI) (n = 2461)	NT-proBNP β (CI) (n = 2413)
Unadjusted model	0.61 (0.46, 0.82)	0.51 (0.33, 0.78)	−0.12 (−0.33, 0.09)	−0.23 (−0.46, 0.00)	0.92 (0.79, 1.06)
Adjusted model	0.70 (0.51, 0.97)	0.52 (0.33, 0.82)	−0.13 (−0.35, 0.10)	−0.18 (−0.41, 0.04)	0.97 (0.83, 1.14)

Panel B. Women					
	Self-rated health OR (CI) (n = 4180)	C-reactive protein OR (CI) (n = 2018)	Cholesterol ratio β (CI) (n = 2505)	Glycated haemoglobin β (CI) (n = 2481)	NT-proBNP β (CI) (n = 2473)
Unadjusted model	0.45 (0.35, 0.59)	0.46 (0.33, 0.64)	−0.42 (−0.56, −0.28)	−0.16 (−0.31, −0.02)	1.01 (0.90, 1.14)
Adjusted model	0.52 (0.39, 0.69)	0.47 (0.33, 0.67)	−0.40 (−0.55, −0.25)	−0.06 (−0.21, 0.09)	1.05 (0.93, 1.19)

Notes: CI = 95% confidence interval; OR = odds ratio. Estimates were obtained from separate regression models for each health outcome and were estimated separately by sex. ORs were estimated using logistic regression for binary outcomes, including SRH and CRP. β coefficients were estimated using linear regression for continuous outcomes, including cholesterol ratio and glycated haemoglobin. NT-proBNP was log-transformed before linear regression; exponentiated coefficients are presented and represent ratios of geometric means.

The direct effect of childhood SEP remained substantial after allowing for mediation through adult SEP, indicating that much of the childhood SEP–health association was not captured by adult SEP measures examined here. Among men (Table 4), for SRH, the interventional direct effects (RR ≈ 0.78–0.87 across mediators) indicated that, when the adult SEP mediator distribution was held fixed, shifting childhood SEP from disadvantaged to advantaged was associated with a 13–22% lower risk of poorer SRH. The interventional indirect effect via occupational class implied that, when childhood SEP was held fixed, shifting the adult SEP distribution from that expected under disadvantaged childhood SEP to that expected under advantaged childhood SEP was associated with a 10% lower risk of poorer SRH (RR = 0.90, 95% CI: 0.86–0.95), corresponding to 39% of the total association mediated. Smaller indirect effects were observed via education (RR = 0.93, 95% CI: 0.90–0.96; 27% mediated), housing (RR = 0.97, 95% CI: 0.94–0.99; 11% mediated), and income (RR = 0.99, 95% CI: 0.97–1.00; 4% mediated).

**Table 4:** Interventional direct and indirect effects of childhood SEP on health, men.

Mediator	Direct effect	Indirect effect	Overall effect	rPM
Self-rated health RR (CI)				
Class	0.87 (0.73, 1.00)	0.90 (0.86, 0.95)	0.79 (0.67, 0.91)	0.39 (0.15, 1.10)
Housing	0.79 (0.69, 0.90)	0.97 (0.94, 0.99)	0.77 (0.67, 0.87)	0.11 (0.03, 0.29)
Education	0.83 (0.73, 0.93)	0.93 (0.90, 0.96)	0.77 (0.67, 0.87)	0.27 (0.10, 0.52)
Income	0.78 (0.68, 0.88)	0.99 (0.97, 1.00)	0.77 (0.67, 0.88)	0.04 (0.00, 0.11)
C-reactive protein RR (CI)				
Class	0.65 (0.50, 0.86)	0.91 (0.83, 0.99)	0.59 (0.46, 0.76)	0.14 (0.01, 0.50)
Housing	0.63 (0.49, 0.85)	1.00 (0.96, 1.03)	0.62 (0.49, 0.84)	–
Education	0.68 (0.54, 0.93)	0.91 (0.86, 0.96)	0.62 (0.49, 0.84)	0.16 (0.07, 0.43)
Income	0.62 (0.49, 0.85)	1.00 (0.99, 1.01)	0.62 (0.49, 0.84)	–
Cholesterol ratio β (CI)				
Class	−0.15 (−0.30, 0.02)	0.00 (−0.05, 0.05)	−0.15 (−0.27, 0.01)	–
Housing	−0.12 (−0.27, 0.02)	−0.01 (−0.04, 0.00)	−0.14 (−0.28, 0.01)	–
Education	−0.10 (−0.24, 0.05)	−0.04 (−0.07, −0.01)	−0.14 (−0.28, 0.01)	–
Income	−0.14 (−0.28, 0.01)	0.00 (−0.01, 0.01)	−0.14 (−0.28, 0.01)	–
Glycated haemoglobin (HbA1c) β (CI)				
Class	−0.10 (−0.22, 0.03)	−0.02 (−0.06, 0.01)	−0.12 (−0.22, −0.01)	–
Housing	−0.14 (−0.24, −0.02)	−0.01 (−0.03, 0.01)	−0.14 (−0.24, −0.03)	–
Education	−0.14 (−0.24, −0.02)	−0.01 (−0.03, 0.02)	−0.14 (−0.24, −0.03)	–
Income	−0.14 (−0.24, −0.04)	0.00 (−0.01, 0.01)	−0.14 (−0.24, −0.03)	–
NT-proBNP β (CI)				
Class	0.03 (−0.07, 0.12)	−0.02 (−0.05, 0.01)	0.01 (−0.09, 0.10)	–
Housing	0.04 (−0.03, 0.14)	0.00 (−0.01, 0.02)	0.04 (−0.03, 0.14)	–
Education	0.04 (−0.04, 0.14)	0.00 (−0.02, 0.03)	0.04 (−0.03, 0.14)	–
Income	0.04 (−0.03, 0.14)	0.00 (−0.01, 0.00)	0.04 (−0.03, 0.14)	–

Notes: RR = risk ratio. CI = 95% confidence interval. rPM = randomised analogue of proportion mediated, quantifying how much of the overall effect is attributable to shifting the mediator distribution. The overall effect is reported for completeness alongside the interventional direct and indirect effects. rPM is reported only when the overall effect is meaningfully different from zero, the interventional direct and indirect effects operate in the same direction, and the indirect effect is sufficiently distinct from the null to support interpretation.

A similar pattern was observed for CRP. Direct effects remained substantial (RR ≈ 0.63–0.68), indicating a strong remaining childhood SEP gradient in elevated CRP after accounting for adult SEP among men. Indirect effects were evident via occupational class and education (RR ≈ 0.91; 14–16% mediated), implying that shifting these adult SEP distributions from those expected under disadvantaged childhood SEP to those expected under advantaged childhood SEP would reduce inflammation risk by around 9%. For cholesterol ratio, HbA1c, and NT-proBNP, interventional indirect effects were close to the null, suggesting that these childhood SEP gradients were largely not captured by adult SEP measures examined here.

Among women (Table 5), direct effects for SRH were also sizeable (RR ≈ 0.66–0.69), indicating that, when the adult SEP mediator distribution was held fixed, advantaged childhood SEP was associated with a 31–34% lower risk of poorer SRH. Indirect effects were smaller than among men and were most evident via education (RR = 0.95, 95% CI: 0.91–0.98; 10% mediated), with limited mediation via housing (6% mediated) and income (3% mediated), and little evidence via occupational class. For CRP, direct effects remained sizeable (RR ≈ 0.62–0.69), with limited mediation, mostly evident via housing (RR = 0.97; 95% CI: 0.92–1.00; 6% mediated). Indirect effects were small or close to the null for cholesterol ratio, HbA1c, and NT-proBNP.

**Table 5:** Interventional direct and indirect effects of childhood SEP on health, women.

Mediator	Direct effect	Indirect effect	Overall effect	rPM
Self-rated health RR (CI)				
Class	0.68 (0.59, 0.80)	0.97 (0.93, 1.01)	0.66 (0.57, 0.78)	–
Housing	0.67 (0.59, 0.77)	0.97 (0.94, 0.99)	0.65 (0.56, 0.75)	0.06 (0.02, 0.12)
Education	0.69 (0.59, 0.79)	0.95 (0.91, 0.98)	0.65 (0.56, 0.75)	0.10 (0.03, 0.20)
Income	0.66 (0.57, 0.76)	0.98 (0.96, 1.00)	0.65 (0.56, 0.75)	0.03 (0.01, 0.08)
C-reactive protein RR (CI)				
Class	0.62 (0.47, 0.77)	1.04 (1.00, 1.09)	0.65 (0.50, 0.80)	–
Housing	0.68 (0.56, 0.81)	0.97 (0.92, 1.00)	0.66 (0.55, 0.79)	0.06 (0.00, 0.18)
Education	0.69 (0.56, 0.82)	0.96 (0.92, 1.01)	0.66 (0.55, 0.79)	–
Income	0.66 (0.54, 0.78)	1.02 (0.99, 1.05)	0.67 (0.56, 0.79)	–
Cholesterol ratio β (CI)				
Class	−0.28 (−0.38, −0.14)	0.00 (−0.03, 0.01)	−0.28 (−0.37, −0.16)	–
Housing	−0.28 (−0.38, −0.15)	−0.01 (−0.03, 0.00)	−0.29 (−0.39, −0.16)	–
Education	−0.29 (−0.39, −0.17)	0.00 (−0.02, 0.02)	−0.29 (−0.39, −0.17)	–
Income	−0.29 (−0.39, −0.17)	0.00 (−0.01, 0.01)	−0.29 (−0.39, −0.16)	–
Glycated haemoglobin (HbA1c) β (CI)				
Class	−0.15 (−0.22, −0.06)	0.00 (−0.02, 0.01)	−0.16 (−0.23, −0.07)	–
Housing	−0.13 (−0.21, −0.06)	−0.01 (−0.02, 0.00)	−0.13 (−0.21, −0.07)	–
Education	−0.13 (−0.22, −0.06)	0.00 (−0.02, 0.01)	−0.13 (−0.22, −0.07)	–
Income	−0.13 (−0.21, −0.07)	0.00 (−0.01, 0.00)	−0.13 (−0.21, −0.07)	–
NT-proBNP β (CI)				
Class	0.04 (−0.05, 0.13)	−0.01 (−0.03, 0.00)	0.03 (−0.06, 0.11)	–
Housing	0.02 (−0.06, 0.08)	0.00 (−0.01, 0.01)	0.02 (−0.05, 0.08)	–
Education	0.03 (−0.05, 0.09)	−0.01 (−0.02, 0.01)	0.02 (−0.05, 0.08)	–
Income	0.02 (−0.06, 0.09)	0.00 (−0.01, 0.01)	0.02 (−0.05, 0.08)	–

Notes: RR = risk ratio. CI = 95% confidence interval. rPM = randomised analogue of proportion mediated, quantifying how much of the overall effect is attributable to shifting the mediator distribution. The overall effect is reported for completeness alongside the interventional direct and indirect effects. rPM is reported only when the overall effect is meaningfully different from zero, the interventional direct and indirect effects operate in the same direction, and the indirect effect is sufficiently distinct from the null to support interpretation.

We undertook a series of post-hoc analyses. These included repeating mediation analyses in the full target population at the most recent sweep (Appendix Tables A6 and A7), testing alternative biomarker specifications (Appendix Tables A8–A10), varying medication-related assumptions (Appendix Tables A11 and A12), restricting analyses to more comparable outcome-collection windows (Appendix Tables A13 and A14) and sample compositions (Appendix Table A15), assessing alternative income operationalisations and time points (Appendix Tables A16–A19), and re-estimating models using alternative childhood SEP indicators, namely housing tenure at age 7 and parental education (Appendix Tables A20–A23). Across these analyses, the substantive pattern of findings was maintained.

## Discussion

Three central findings emerged from our analyses. First, childhood SEP remained associated with later-life health more than six decades later, most consistently for SRH and CRP. Cholesterol-ratio associations were clearer among women, whereas associations with HbA1c and NT-proBNP were weak in both sexes. Second, mediation through adult SEP was modest and outcome-specific: adult SEP accounted for some share of the association for SRH and CRP, but little of the association for cardiometabolic biomarkers. Third, mediation differed by adult SEP indicators and sex: occupational class and education explained more among men, whereas stronger overall associations among women were less well captured by the adult SEP measures included.

Under a hypothetical equalisation of adult SEP, a substantial childhood SEP gradient in health remained, particularly for SRH and CRP. These findings align with developmental origins and life-course epidemiological frameworks, which emphasise early-life material and psychosocial conditions as foundational determinants of later-life health.^1^^,^^2^ Mediation was outcome specific. Indirect effects were more evident for SRH and CRP, whereas those for cholesterol ratio, HbA1c, and NT-proBNP were close to the null, despite persisting overall associations for some biomarkers. These findings suggest that adult SEP captured only part of the pathways linking childhood SEP to later-life health. One possible explanation is that occupational class, education, housing tenure, and income do not fully represent the wider set of resources through which socioeconomic advantage influences health across the life course. Fundamental cause theory highlights such flexible resources—including knowledge, power, material capacity, and social connections—which can be deployed to protect health as risks and opportunities change over time.^28^ The substantial interventional direct effects therefore should be interpreted as the remaining childhood SEP–health association not captured by adult SEP as measured and modelled here and may partly reflect incomplete measurement or specification of the mediators, or mediation through pathways not examined in this study, rather than wholly unmediated effects.

Our findings are consistent with a broader life-course literature documenting that both childhood and adult SEP matter for later-life health.^6^ Earlier NCDS studies reported independent and comparable associations of childhood and adult SEP with midlife health in mutually adjusted models.^7^ By extending follow-up into early older age and formally quantifying mediation through four dimensions of adult SEP, we found that adult SEP mediated some of the childhood SEP gradient in SRH and, to a lesser extent, CRP, but explained little of the association with cardiometabolic biomarkers. This suggests that adult SEP may play a more limited mediating role in early older age than might be inferred from midlife studies based on informal mediation approaches. Consistent with this interpretation, mutually adjusted models in our data showed only modest attenuation of the childhood SEP associations after adjustment for adult SEP (results available upon request), indicating that the limited mediation is unlikely to be an artefact of the interventional framework and instead reflects the underlying pattern of associations at this later life-course stage.

Two potential explanations may account for the weaker mediating role of adult SEP in our study. First, by early older age, cardiometabolic risk may increasingly reflect the long-term biological embedding of childhood disadvantage. Early-life adversity may contribute to cumulative physiological wear and tear that becomes more apparent with age and may not be easily offset by later socioeconomic circumstances. Adult SEP may therefore remain relevant, but account for a smaller share of childhood SEP gradients in early older age than might be expected from midlife studies. This interpretation should also be considered alongside differences in SEP measures, outcomes, and analytical approaches across studies, which limit direct comparability. Second, the later life-course stage of our analysis may also matter. Socioeconomic differences in survival may begin to narrow socioeconomic contrasts by early older age, potentially reducing the apparent mediating role of adult SEP. However, because outcomes were measured at ages 60–66, such processes are likely to be only emerging and are unlikely to fully explain the limited mediation observed.

The contrasting pattern across outcomes may be informative. A larger share of the childhood SEP gradient in SRH was captured by adult SEP, whereas among objective biomarkers, mediation was evident only to a limited extent for CRP and was minimal for cardiometabolic markers. One plausible explanation is that SRH is a broad evaluative construct encompassing physical functioning, psychological wellbeing, and social comparison, and that the dimensions individuals consider when rating their health may vary across social groups.^19^^,^^29^ This may make SRH more responsive to adult SEP measured close to outcome assessment. In contrast, cardiometabolic markers reflect longer-term physiological embedding of early-life conditions.^14^ This interpretation is consistent with previous evidence of mediation through adult SEP for mental health, including findings from the same cohort.^30^ Together, these findings suggest that the life-course processes linking childhood SEP to later health differ across health domains. While differences in outcome measurement and operationalisation may have influenced the magnitude of these differences, they are unlikely to fully account for the overall pattern observed. Future research should extend this framework to a wider range of outcomes and repeated health measurements to examine how socioeconomic inequalities emerge and evolve differently across health domains, and whether the contribution of adult SEP changes across the life course.

Mediation patterns also varied by adult SEP indicators and sex. Among men, occupational class and education accounted for the largest indirect effects, particularly for SRH and CRP, suggesting that labour-market position and educational attainment were important pathways linking childhood SEP to later health. Among women, mediation was weaker overall: occupational class explained little of the association, while education and housing tenure accounted for only a modest share, leaving most of the childhood SEP–health association not captured by the adult SEP measures considered. This may reflect gendered socioeconomic trajectories in the 1958 cohort. Men’s occupational class more often reflected continuous full-time employment and may have served as a proxy for cumulative adult socioeconomic exposure. For women, occupational class may have captured adult material and social circumstances less completely because of part-time work, labour-force interruptions, and weaker labour-market attachment.^31^ Housing tenure, as a household-level indicator, may therefore have better reflected the economic context in which many women in this generation lived.

Several limitations should be acknowledged. We operationalised childhood and adult SEP using broad binary categories to provide interpretable contrasts and maintain adequate support for the mediation analyses. Although this may have reduced within-category variation and left residual confounding, sensitivity analyses using alternative income thresholds produced substantively similar findings (Appendix Tables A16 and A17). Although the interventional mediation framework strengthens causal interpretation relative to conventional mediation approaches, it relies on the assumption that important confounders of the exposure–outcome, exposure–mediator and mediator–outcome relationships have been adequately captured. While we adjusted for a broad range of childhood and adult characteristics, some residual confounding from unmeasured genetic, early-life, or long-term psychosocial and environmental factors cannot be excluded. In addition, the adult SEP indicators examined and modelled here may not fully capture the socioeconomic and related life-course pathways linking childhood SEP to later-life health. Although substantial attrition is inevitable in a cohort followed for more than six decades, sensitivity analyses accounting for sweep non-response produced highly similar findings (Appendix Tables A6 and A7), suggesting that selective loss to follow-up is unlikely to have materially affected the estimated mediation effects. Nevertheless, some residual selection bias due to attrition on unmeasured factors cannot be excluded. Finally, these findings derive from a single cohort born in Britain in 1958. The extent and pattern of mediation may differ across generations and settings with different socioeconomic conditions and institutional contexts and should therefore be examined in other populations.

Notwithstanding these limitations, this study extends life-course epidemiology and ageing research by applying an interventional mediation framework to multiple socioeconomic dimensions and health outcomes in early older age. Drawing on more than six decades of prospective data from the NCDS, we quantified how childhood SEP remained associated with health at ages 60–66 and the extent to which this association operated through four distinct dimensions of adult SEP. By applying an interventional mediation framework, we moved beyond conventional approaches to estimate policy-relevant “what if” contrasts. These contrasts suggest that population-level improvements in adult SEP could reduce some later-life health inequalities associated with childhood disadvantage but are unlikely to eliminate them. In practice, the most realistic levers for shifting adult SEP at scale are population-level policies affecting financial and employment conditions, alongside early-life interventions to reduce disadvantage. The policy relevance of these hypothetical contrasts depends on the extent to which comparable shifts in adult SEP are feasible within the prevailing social and policy context.^32^

## Contributors

Y.G. conceptualization, methodology, performed the statistical analyses, wrote the first draft, review and editing. A.P. conceptualization, interpretation of the results, supervision, review and editing. G.B.P. conceptualization, methodology, interpretation of the results, supervision, review and editing. A.G. conceptualization, interpretation of the results, supervision, review and editing. Y.G. and G.B.P. have directly accessed and verified the underlying data reported in the manuscript.

## Data sharing statement

All original datasets from the 1958 NCDS are freely available to download at the UK Data Archive.

## Declaration of interests

We declare no competing interests.

## Appendix A Supplementary data

Appendix Fig. A1, Texts A1–A3, and Tables A1–A5
