# Supplementary material for: Life-course socioeconomic position and health in older adulthood: a causal mediation analysis in the 1958 British Birth Cohort
Source: Lancet Reg Health Eur. 2026 Oct 15;71:101890. doi: 10.1016/j.lanepe.2026.101890 (PMC13694844; doi:10.1016/j.lanepe.2026.101890)
Supplement: Supplementary file 1 [file mmc1.docx]

**Life-course socioeconomic position and health in older adulthood: a formal mediation analysis in the 1958 British Birth Cohort**

**Appendix**

**Content**

Table A1 Prospective evidence on childhood SEP–health associations and the role of adult SEP

Table A2 Self-rated health sample characteristics based on available data

Table A3 Biomarker sample characteristics based on available data

Figure A1 Derivation of the analytical sample

Text A1 Corrections for medication use

Text A2 Interventional causal mediation analysis

Text A3 Missing data strategy

Table A4 The proportion of missing information in each variable in the SRH sample

Table A5 The proportion of missing information in each variable in the biomarker sample

Sensitivity analysis

A. Mediation analyses in the full target population at the most recent sweep

- Estimates for men — Table A6
- Estimates for women — Table A7

B. Alternative outcome specifications

- CRP modelled continuously — Table A8
- **Cholesterol ratio** modelled as **binary** — Table A9
- **HbA1c** modelled **as binary** — Table A10

C. Alternative medication assumptions

- Asumed 20% and 30% reductions in total cholesterol among statin users — Table A11
- Excluding participants on heart-failure medications for NT-proBNP — Table A12

D. Sample restrictions

- Biomarker analyses restricted to measurements collected in 2022–2023 — Table A13–A14
- Self-rated health analyses restricted to blood-sample providers — Table A15

E. Income mediation robustness checks

- Income mediation using alternative income operationalisations — Tables A16–A17
- **Income mediation using income measured at alternative time points** — Table A18– A19

F. Alternative indicators of childhood SEP

- Housing tenure at age 7 as the childhood SEP exposure— Tables A20–A21
- Parental education as the childhood SEP exposure — Tables A22–A23

**Table A1** Prospective evidence on childhood SEP–health associations and the role of adult SEP

| Study | Cohort, age at outcome | Health outcome(s) | Exposure | Adult SEP considered? how (analytical approach) | Main finding regarding the role of adult SEP |
| --- | --- | --- | --- | --- | --- |
| Power & Matthews 1997, *Lancet* 350(9091):1584–1589 | 1958 NCDS at 33 | Behavioral, psychological, physiological | Social class at birth (father's occupation) | Minimal. Focus on social origins driving accumulation of risk across the life course; adult SEP not separately modeled. |  |
| Power et al. 2007, *Int J Epidemiol* 36(3):532–539 | 1958 NCDS at 44/45 | Biomarkers and functional outcomes | Social class at birth (father's occupation) | Yes, via mutual adjustment; child and adult class entered together to show childhood effects are independent of adult position. | Childhood and adult social class both contributed to midlife biomarkers/functional outcomes in mutually adjusted models |
| Tabassum et al. 2008, *Am J Epidemiol* 167(11):1332–1341 | 1958 NCDS at 44/45 | Inflammatory and hemostatic markers | Life-course SEP, social class at birth, age 23/42, combined into a cumulative indicator score | Yes, but as part of a cumulative accumulation model. Adult SEP is summed with childhood SEP into a single lifetime score rather than modelled separately. |  |
| Chen & Lacey 2018, *Brain Behav Immun* 69:582–590 | 1958 NCDS at 44/45 | C-reactive protein and fibrinogen | Adverse childhood experiences (ACE score), measured prospectively across ages 0 to 16 | Yes, but as a mediator via sequential adjustment. The ACE to CRP association attenuated after adding socioeconomic factors and disappeared once all three were included, inferred as mediation | Adult socioeconomic factors appeared to partly explain the association between adverse childhood experiences and adult inflammation. |
| Rogers et al. 2021, *Am J Epidemiol* 190(8):1550–1560 | 1958 NCDS at 50; 50-58 | A 34-item frailty index (accumulation of health-related deficits); mortality | Social class at birth (father's occupation) | Yes, formal counterfactual mediation of the frailty index: total effect split into natural direct and natural indirect effects through adult SEP (single latent variable), via g-computation with imputation |  |
| Hurst et al. 2013, *Am J Public Health* 103(9):1641–1648 | 1946 NSHD at 60/64 | Objectively assessed physical and cognitive functioning | Social class at 4 (father's occupation) and adult occupational class at 53 | Yes, but via mutual adjustment assessing the relative importance of childhood versus adult SEP, no formal counterfactual mediation decomposition |  |
| Bann et al. 2014, *J Epidemiol Community Health* 68(6):516–523 | 1946 NSHD at 60/64 | DXA-measured body composition: fat mass index, android-to-gynoid fat ratio, and lean mass | Paternal occupational class at 4 and education; household occupational class at 53 and income at 60–64 | Yes, but as an explanatory factor via adjustment. Tested whether childhood SEP associations were explained by adult SEP (alongside pre-adulthood weight gain); found them partly explained. | Adult SEP partly explained childhood SEP differences in later-life body composition. |
| Bann et al. 2015, *Soc Sci Med* 147:190–199 | 1946 NSHD at 53 and 60 to 64 | Endocrine markers across multiple axes: free testosterone and IGF-I, evening cortisol, and a composite adverse hormone score | Paternal occupational class at age 4, education at 26, household occupational class at 53, household income at 60 to 64 | Yes, but as a life-course indicator comparison, not mediation. Each SEP indicator (childhood, education, midlife, later-life) was related to each hormone in its own regression to see which life-stage indicator mattered most. | The study did not frame adult SEP as a mediator of childhood SEP. |
| Kok et al. 2019, *J Epidemiol Community Health* 73(6):529–536 | 1946 NSHD at 60/64 | Self-reported functional limitations (difficulty with at least 1 of 16 daily activities) | Lifetime socioeconomic adversity: latent-class trajectories over ages 4 to 53 | Not as a mediator. Adult SEP is folded into the lifetime adversity trajectory (the exposure itself). | The study did not frame adult SEP as a mediator of childhood SEP. |
| **Kuh et al. 2002, *BMJ* 325:1076–80.** | 1946 NSHD at 26/54 | Premature mortality | Father's occupational class at 4, housing conditions, quality of childhood care | Yes, but via mutual adjustment. Adult SEP at 26 (especially home ownership) entered alongside childhood indicators; manual origins and poor childhood care remained associated with mortality after adjustment. |  |
| Murray et al. 2011, *Ann Epidemiol* 21(8):589–97 | NSHD at 53 | Cardiometabolic risk factors: BMI, SBP, DBP, total cholesterol, LDL, HDL, triglycerides, HbA1c | Occupational SEP at childhood, early adulthood and middle age (life-course) | Partly, via life-course model comparison. Adult SEP is a component of these models rather than a separate mediator. | The study did not frame adult SEP as a mediator of childhood SEP. |
| Murray et al. 2016, *PLoS ONE* 11(3):e0152691 | NSHD at 60/64 | Echocardiographic markers: LV mass index, relative wall thickness, ejection fraction, midwall fractional shortening, left atrial volume, E/A and E/e' ratios | Occupational SEP at childhood, early adulthood and middle age (life-course) | Partly, via life-course model comparison. Adult SEP is a component of these models rather than a separate mediator. | The study did not frame adult SEP as a mediator of childhood SEP. |
| Khanolkar et al. 2021, *PLoS Med* 18(9): e1003775 | NSHD at 36/69 | Multimorbidity (count of 18 conditions from self-report, biomarkers, health records and prescriptions) and its trajectory | Childhood social class, plus adult social class and adult education | Yes, but via mutual adjustment. Childhood and adult SEP indicators entered simultaneously; disadvantaged childhood social class was associated with accelerated multimorbidity trajectories from age 53 independently of adult SEP. | Adult SEP was associated with multimorbidity trajectories, but disadvantaged childhood social class remained linked to accelerated multimorbidity independently of adult SEP. |
| **Barboza Solís et al. 2016,** *Soc Sci Med* 165:19–27 | NCDS at 44 | Allostatic load, a composite of 14 biomarkers across four physiological systems | Parental SEP at birth (maternal education and parental occupation) | Yes, via path analysis. Parental SEP to allostatic load decomposed into educational, material and health-behaviour paths, the educational path dominated. Product-of-coefficients path mediation. |  |
| Winpenny et al. 2021, *J Epidemiol Community Health* (doi:10.1136/jech-2021-216611) | BCS70 at 46 | Cardiometabolic risk factors: waist circumference, SBP, DBP, triglycerides, HDL-c, non-HDL-c, HbA1c, CRP | Early-adulthood socioeconomic trajectories (ages 16 to 24), six latent classes. | Yes, adult SEP (social class and income at 46) modelled as two parallel mediators in an Mplus path model, decomposing the total effect into direct and indirect (product-of-coefficients) paths per outcome. Indirect paths were small and mostly null. | Adult social class and income at 46 generally showed small or mostly null indirect pathways between early-adulthood SEP trajectories and cardiometabolic outcomes. |
| Wood et al. 2017, *PLoS ONE* 12(10): e0185798 | Four British cohorts: HCS/NSHD/NCDS/BCS70 at 73/60–64/50/42 | Mental wellbeing (Warwick-Edinburgh Mental Wellbeing Scale) | Childhood SEP (father's Registrar General's social class at 10/11) | Yes, but tested as a mediator via multi-group path analysis (SEM). Mediation of the childhood-SEP to wellbeing association through own education and adult social class; in NCDS the association was fully mediated by adult SEP, in BCS70 partially (direct plus indirect). | Adult SEP, especially education and adult social class, mediated the association between childhood SEP and mental wellbeing; mediation appeared stronger in NCDS than in BCS70. |
| Robertson, Popham & Benzeval 2014, *BMC Public Health* 14, 184 | West of Scotland Twenty-07 Study; respondents aged 35, 55 and 75 | Allostatic load, based on nine cardiovascular, metabolic and inflammatory biomarkers | SEP across childhood, transition to adulthood and adulthood | Yes, but as part of life-course model comparison rather than mediation. |  |
| Robertson et al. 2015, *Brain, Behavior, and Immunity,* ([10.1016/j.bbi.2014.10.005](https://doi.org/10.1016/j.bbi.2014.10.005)) | West of Scotland Twenty-07 Study; allostatic load measured at age 55 | Allostatic load, combining cardiovascular, metabolic and inflammatory markers | Accumulated social class across adulthood, measured over two periods from ages 35 to 55 | Yes, but not specifically as adult SEP mediation of childhood SEP. The study examined material, psychosocial and behavioural mediators of the SEP–allostatic-load association using path analysis / attenuation logic. |  |
| Lawlor et al. 2006, *American Journal of Public Health,* PMCID: [PMC1483862](https://pmc.ncbi.nlm.nih.gov/articles/PMC1483862/) | Aberdeen Children of the 1950s cohort; adult follow-up for fatal and non-fatal CVD | Fatal and non-fatal cardiovascular disease | Social class at birth | Partly. Educational attainment was examined as an explanatory pathway, but adult SEP was not formally decomposed as a mediator in a counterfactual framework. |  |
| Hossin, Koupil & Falkstedt 2019, *BMJ Open,* [10.1136/bmjopen-2018-026258](https://doi.org/10.1136/bmjopen-2018-026258?urlappend=%3Futm_source%3Dresearchgate.net%26utm_medium%3Darticle) | Stockholm Public Health Cohort; participants aged over 40, followed for mortality 2002–2011 | CVD mortality; non-CVD mortality as comparison | Father’s SEP, manual versus non-manual | Yes, formal causal mediation. Adult social mediators included own education and adult SEP; behavioural mediators included smoking, alcohol, physical inactivity, diet and BMI. Inverse odds weighting was used to estimate natural direct and indirect effects. |  |
| Mishra et al. 2013 / Heshmati et al. 2020, Longitudinal and Life Course Studies, 11(1) | Uppsala Birth Cohort Multigenerational Study; mortality follow-up into later life | All-cause mortality and circulatory disease mortality | SEP measured at birth, childhood, adulthood and later life | Yes, but as life-course SEP indicators rather than a mediator. Compared critical-period, sensitive-period and accumulation models using Cox models. |  |
| Hossin et al. 2022, *European Journal of Public Health,* 32(4) | Uppsala Birth Cohort Multigenerational Study; cohort born 1915–1929, followed for mortality | All-cause and cause-specific mortality, including CVD mortality | Latent SEP trajectories across birth, childhood, mid-adulthood and late adulthood | Yes, but as part of latent life-course SEP trajectories. Adult SEP was incorporated into trajectory classes rather than isolated as a mediator. |  |
| Gurzo et al. 2022, *International Journal of Environmental Research and Public Health,* 19(12) | Stockholm Birth Cohort Multigenerational Study; 1953 Stockholm cohort, mortality follow-up ages 48–68 | All-cause mortality | Childhood parental income | Yes, but framed as intermediate pathways rather than formal mediation. Adult educational attainment, occupational status and adult income were included alongside cognitive ability and social skills. |  |

**Table A2** Self-rated health sample characteristics based on available data

|  | Men | | Women | |
| --- | --- | --- | --- | --- |
| Variable | N | Mean or % | N | Mean or % |
| Self-rated health |  |  |  |  |
| Good | 3,095 | 74.0 | 3,098 | 74.1 |
| Fair/poor | 1,088 | 26.0 | 1,082 | 25.9 |
| Total | 4,183 |  | 4,180 |  |
| Childhood social class |  |  |  |  |
| Manual | 2,579 | 67.7 | 2,586 | 68.6 |
| Non-manual | 1,230 | 32.3 | 1,183 | 31.4 |
| Total | 3,809 |  | 3,769 |  |
| Housing tenure at 7 |  |  |  |  |
| Rented | 1,912 | 52.7 | 1,982 | 54.0 |
| Owned | 1,719 | 47.3 | 1,688 | 46.0 |
| Total | 3,631 |  | 3,670 |  |
| Parental education |  |  |  |  |
| Neither parent remained to age 16 | 1,992 | 64.8 | 2,020 | 64.2 |
| At least one parent remained to age 16 or older | 1,081 | 35.2 | 1,126 | 35.8 |
| Total | 3,073 |  | 3,146 |  |
| Social class at 55 |  |  |  |  |
| Manual | 1,164 | 37.5 | 670 | 23.3 |
| Non-manual | 1,939 | 62.5 | 2,203 | 76.7 |
| Total | 3,103 |  | 2,873 |  |
| Economic activity at 55 |  |  |  |  |
| Inactive | 471 | 13.0 | 839 | 22.0 |
| Active | 3,166 | 87.0 | 2,971 | 78.0 |
| Total | 3,637 |  | 3,810 |  |
| Housing tenure at 55 |  |  |  |  |
| Renter | 397 | 19.3 | 458 | 19.0 |
| Owner | 1,657 | 80.7 | 1,953 | 81.0 |
| Total | 2,054 |  | 2,411 |  |
| Education at 55 |  |  |  |  |
| Non-degree qualifications | 2,727 | 76.2 | 2,881 | 76.7 |
| Degree or higher university degree | 850 | 23.8 | 876 | 23.3 |
| Total | 3,577 |  | 3,757 |  |
| Income at 55 (log transformed) | 2,802 | 10.1 | 2,618 | 10.0 |
| Age at assessment, mean (SD) |  | 63.6 (1.4) |  | 63.6 (1.4) |
| Age at assessment |  | 60–66 |  | 60–66 |
| 60 | 29 | 0.69 | 29 | 0.69 |
| 61 | 590 | 14.05 | 643 | 15.29 |
| 62 | 193 | 4.60 | 178 | 4.23 |
| 63 | 418 | 9.95 | 455 | 10.82 |
| 64 | 2,185 | 52.04 | 2,202 | 52.35 |
| 65 | 469 | 11.17 | 418 | 9.94 |
| *Table A2 continued* | | | | |
| 66 | 315 | 7.50 | 281 | 6.68 |
| Child’s ethnicity |  |  |  |  |
| Not BME | 4,107 | 97.8 | 4,112 | 97.8 |
| BME | 92 | 2.2 | 94 | 2.2 |
| Total | 4,199 |  | 4,206 |  |
| Grandparental class |  |  |  |  |
| Manual | 2,359 | 71.8 | 2,371 | 71.7 |
| Non-manual | 927 | 28.2 | 936 | 28.3 |
| Total | 3,286 |  | 3,307 |  |
| Smoking |  |  |  |  |
| No | 2,514 | 71.3 | 2,654 | 71.2 |
| Yes | 1,011 | 28.7 | 1,072 | 28.8 |
| Total | 3,525 |  | 3,726 |  |
| Regular alcohol use |  |  |  |  |
| No | 970 | 27.5 | 1,928 | 51.7 |
| Yes | 2,563 | 72.5 | 1,803 | 48.3 |
| Total | 3,533 |  | 3,731 |  |
| Low exercise |  |  |  |  |
| No | 2,807 | 79.7 | 2,946 | 79.3 |
| Yes | 717 | 20.3 | 770 | 20.7 |
| Total | 3,524 |  | 3,716 |  |
| Having long-standing illness or not |  |  |  |  |
| No | 2,943 | 83.4 | 3,225 | 86.5 |
| Yes | 585 | 16.6 | 502 | 13.5 |
| Total | 3,528 |  | 3,727 |  |
| Marital status |  |  |  |  |
| Married | 2,435 | 71.1 | 2,700 | 73.8 |
| Not married | 989 | 28.9 | 961 | 26.2 |
| Total | 3,424 |  | 3,661 |  |
| Malaise score (standardized) | 3,528 | 0.2 | 3,723 | -.0.1 |
| Number of child | 3,201 | 1.4 | 3,495 | 1.6 |
| N in Main Survey | 4,199 |  | 4,206 |  |

**Table A3** Biomarker sample characteristics based on available data

|  | Men | | Women | |
| --- | --- | --- | --- | --- |
| Variable | N | Mean or % | N | Mean or % |
| C-reactive protein (CRP) |  |  |  |  |
| Presence of inflammation | 374 | 18.7 | 512 | 25.4 |
| No inflammation | 1,622 | 81.3 | 1,506 | 74.6 |
| Total | 1,996 |  | 2,018 |  |
| HDL cholesterol (mmol/L) | 2,479 | 1.4 | 2,507 | 1.7 |
| Total cholesterol (mmol/L) | 2,484 | 5.7 | 2,506 | 6.1 |
| Cholesterol ratio | 2,479 | 4.3 | 2,505 | 3.8 |
| Glycated hemoglobin (HbA1c) (%) | 2,461 | 6.1 | 2,481 | 6.0 |
| NT-proBNP (pg/mL) (log transformed) | 2,413 | 4.0 | 2,473 | 4.3 |
| Childhood social class |  |  |  |  |
| Manual | 1,930 | 67.3 | 1,925 | 67.8 |
| Non-manual | 937 | 32.7 | 913 | 32.2 |
| Total | 2,867 |  | 32.2 |  |
| Housing tenure at 7 |  |  |  |  |
| Renter | 1,444 | 52.3 | 1,471 | 53.1 |
| Owner | 1,315 | 47.9 | 1,298 | 46.7 |
| Total | 2,759 |  | 2,769 |  |
| Parental education |  |  |  |  |
| Neither parent remained to age 16 | 1,514 | 64.8 | 1,532 | 63.7 |
| At least one parent remained to age 16 or older | 822 | 35.2 | 875 | 36.3 |
| Total | 2,336 |  | 2,407 |  |
| Social class at 55 |  |  |  |  |
| Manual | 886 | 36.3 | 527 | 23.1 |
| Non-manual | 1,556 | 63.7 | 1,759 | 76.9 |
| Total | 2,442 |  | 2,286 |  |
| Economic activity at 55 |  |  |  |  |
| Inactive | 354 | 12.5 | 610 | 20.5 |
| Active | 2,484 | 87.5 | 2,361 | 79.5 |
| Total | 2,838 |  | 2,971 |  |
| Housing tenure at 55 |  |  |  |  |
| Renter | 289 | 18.4 | 322 | 17.4 |
| Owner | 1,282 | 81.6 | 1,527 | 82.6 |
| Total | 1,571 |  | 1,849 |  |
| Education at 55 |  |  |  |  |
| Non-degree qualifications | 2,122 | 75.1 | 2,241 | 75.8 |
| Degree or higher university degree | 702 | 24.9 | 714 | 24.2 |
| Total | 2,824 |  | 2,955 |  |
| Income at 55 (log transformed) | 2,250 | 10.1 | 2,100 | 10.1 |
| Age at assessment, mean (SD) |  | 63.9 (1.3) |  | 63.9 (1.3) |
| Age at assessment |  | 60–65 |  | 60–65 |
| 60 | 4 | 0.13 | 4 | 0.13 |
| *Table A3 continued* | | | | |
| 61 | 351 | 11.15 | 354 | 11.20 |
| 62 | 127 | 4.03 | 132 | 4.18 |
| 63 | 72 | 2.29 | 77 | 2.44 |
| 64 | 1,349 | 42.85 | 1,355 | 42.87 |
| 65 | 1,245 | 39.55 | 1,239 | 39.20 |
| Child’s ethnicity |  |  |  |  |
| Not BME | 3,089 | 98.1 | 3,088 | 97.7 |
| BME | 59 | 1.9 | 73 | 2.3 |
| Total | 3,148 |  | 3,161 |  |
| Grandparental class |  |  |  |  |
| Manual | 1,777 | 71.5 | 1,772 | 71.3 |
| Non-manual | 707 | 28.5 | 712 | 28.7 |
| Total | 2,484 |  | 2,484 |  |
| Smoking |  |  |  |  |
| No | 2,011 | 73.3 | 2,094 | 72.8 |
| Yes | 731 | 26.7 | 782 | 27.2 |
| Total | 2,742 |  | 2,876 |  |
| Regular alcohol use |  |  |  |  |
| No | 772 | 28.1 | 1,472 | 51.1 |
| Yes | 1,977 | 71.9 | 1,408 | 48.9 |
| Total | 2,749 |  | 2,880 |  |
| Low exercise |  |  |  |  |
| No | 2,208 | 80.6 | 2,276 | 79.3 |
| Yes | 533 | 19.4 | 594 | 20.7 |
| Total | 2,741 |  | 2,870 |  |
| Having long-standing illness or not |  |  |  |  |
| No | 2,298 | 83.7 | 2,489 | 86.5 |
| Yes | 447 | 16.3 | 389 | 389 |
| Total | 2,745 |  | 2,878 |  |
| Marital status |  |  |  |  |
| Married | 1,924 | 72.2 | 2,093 | 74.1 |
| Not married | 742 | 27.8 | 732 | 25.9 |
| Total | 2,666 |  | 2,825 |  |
| Malaise score (standardized) | 2,746 | 0.2 | 2,877 | -0.0 |
| Number of child | 2,487 | 1.4 | 2,696 | 1.6 |
| N in Main Survey | 3,148 |  | 3,161 |  |

**Figure A1** Derivation of the analytical sample

Notes: Models specifying adult occupational class as the mediator additionally excluded participants out of the labour force at age 55, reducing samples to: self-rated health 3,715 men / 3,347 women; CRP 1,787 / 1,649; cholesterol ratio 2,220 / 2,034; HbA1c 2,204 / 2,015; NT-proBNP 2,155 / 2,014.

**Text A1** Corrections for medication use

Several cardiometabolic biomarkers examined in this study are influenced by pharmacological treatment. We therefore applied an add-back correction to approximate untreated biomarker levels among participants using relevant medications. The add-back approach assumes treatment lowers biomarker values by an approximately known average amount and adds this amount back to treated individuals to approximate their underlying untreated levels. This method was formalised by Tobin et al. (2005) and is widely used in observational epidemiology for biomarkers affected by medication (Choi et al., 2022).

The cholesterol ratio is influenced primarily by lipid-lowering medications, which reduce total cholesterol but have modest effects on HDL cholesterol. Meta-analyses of statin trials suggest total cholesterol reductions of roughly 17–35% depending on drug and dose (Edwards & Moore, 2003; Law et al., 2003). Following prior observational work, we assumed an average 25% reduction in total cholesterol among statin users. Therefore, for participants reporting lipid-lowering medication, untreated total cholesterol was estimated by dividing the observed value by 0.75; HDL cholesterol was not corrected. The cholesterol ratio was computed using corrected total cholesterol and observed HDL cholesterol.

HbA1c reflects long-term glycaemic control and is directly affected by glucose-lowering medications. Evidence suggests treatment reduces HbA1c by around 0.8–1.5 percentage points on average and adding 1 percentage point is commonly used as a pragmatic correction (Tobin et al., 2005; Eastwood et al., 2016). Accordingly, for participants reporting diabetes medication, we added 1 percentage point to observed HbA1c. NT-proBNP is not a direct treatment target and may be influenced by multiple cardiovascular medication classes with heterogeneous, severity-dependent effects (Ponikowski et al., 2016; Yancy et al., 2017; Januzzi et al., 2018). As no widely accepted correction exists, NT-proBNP was analysed using observed values only.

**Text A2** Interventional causal mediation analysis

**Notation and estimands.** Let A denote childhood social class, coded a = 1 for non-manual and a* = 0 for manual; M an adult SEP mediator; and Y a health outcome. Baseline confounders C precede the exposure; exposure-induced confounders L (Figure 1). All analyses were conducted separately for men and women. Causal effects are defined through potential outcomes Y(a, m), the outcome that would be observed under childhood social class a and adult SEP value m.

Interventional effects are defined through population-level interventions on the mediator. Let M(a | C) denote a random draw from the distribution of the adult SEP mediator within strata of the baseline confounders C under childhood social class A = a. Intuitively, each individual's mediator is not fixed to a single value but is drawn at random from the distribution observed among people who share the same baseline characteristics and the same level of childhood social class. On the difference scale, the interventional direct (IDE) and indirect effects (IIE) are:

IDE = E[Y(a, M(a* | C))] − E[Y(a*, M(a* | C))]

IIE = E[Y(a, M(a | C))] − E[Y(a, M(a* | C))]

The interventional direct effect contrasts non-manual (advantaged) versus manual (disadvantaged) childhood social class while holding the adult SEP distribution fixed at the level it would take under manual (disadvantaged) childhood social class. The interventional indirect effect holds childhood SEP fixed at the non-manual (advantaged) level and compares outcomes under the adult SEP distribution expected under non-manual (advantaged) childhood SEP with outcomes under the adult SEP distribution expected under manual (disadvantaged) childhood SEP. Their sum is the overall effect:

OE = E[Y(a, G(a | C))] − E[Y(a*, G(a* | C))]

which is the randomised analogue of the total effect. Under exposure-induced (intermediate) confounding the interventional effects decompose this overall effect rather than the total effect, and we therefore report the overall effect alongside the direct and indirect effects. To summarise the share of the overall effect operating through adult SEP, we report the randomised analogue of the proportion mediated, rPM = IIE / OE. We interpret rPM only when the overall effect is meaningfully different from zero and the direct and indirect effects operate in the same direction.

**Drawing the mediator within strata of C.** We draw the mediator from its distribution within strata of the baseline confounders C rather than from its marginal population distribution. This corresponds to a more realistic hypothetical intervention, since an intervention on adult SEP would plausibly affect individuals differently according to their baseline characteristics rather than reassign the mediator uniformly. It also weakens the positivity requirement, which then needs to hold only within strata of C rather than across the whole population.

**Identification assumptions. Identification of the interventional direct and indirect effects requires conditional exchangeability: no unmeasured confounding of the childhood social class–health and childhood social class–adult SEP relationships given baseline covariates C, and no unmeasured confounding of the adult SEP–health relationship given childhood social class, C, and the measured intermediate confounders L. It also requires positivity—that each level of childhood social class and the relevant adult SEP values or distributions have sufficient support within the corresponding covariate strata—together with consistency, whereby observed mediator and outcome values correspond to their counterfactual values under the exposure and mediator levels actually experienced, and no interference between cohort members. Interventional effects replace each individual’s unobservable counterfactual mediator value with a random draw from the population distribution of the mediator under each exposure level within strata of C.** Unlike natural direct and indirect effects, interventional effects do not require the cross-world independence assumption and therefore remain identified in the presence of exposure-induced mediator–outcome confounding, provided the intermediate confounders L are measured. This property motivated our use of the interventional framework, since the age 33 behavioral, health, and social factors are plausibly affected by childhood social class while also confounding the adult SEP–health relationship, a setting in which natural effects would not be identified.

**Estimation.** We estimated the interventional effects using g-computation with direct counterfactual imputation, implemented in the CMAverse R package with the CMAversePlus add-on. In brief, a model for the mediator given A, C, and L, and a model for the outcome given A, M, L, and C, were fitted and used to simulate the potential outcomes entering each estimand. The mediator was drawn from its conditional distribution as described above, and the intermediate confounders were accommodated by averaging over their distribution given childhood social class and baseline confounders. 95% confidence intervals were obtained using non-parametric bootstrapping, with missing data handled through multiple imputation within each bootstrap sample (Boot MI).

**Text A3** Missing data strategy

Loss to follow-up is unavoidable in longitudinal studies. Previous work using the NCDS has shown that non-response is systematically related to observed characteristics (Mostafa et al., 2020). To address item non-response, we imputed missing values using multivariate imputation by chained equations (MICE), generating 50 imputed datasets. Multiple imputation assumes data are Missing At Random (MAR), that is, that the observed data can account for systematic differences between observed and missing values (White, Royston & Wood, 2011); this assumption is largely untestable but can be made more plausible by including variables predictive of non-response and of the underlying missing values. To make the MAR assumption more plausible, our imputation models included all variables in the substantive models together with a diverse set of auxiliary variables predictive of non-response or of the underlying missing values: mother's age at age 0, number of persons per room at age 0, father's education, social problems (e.g. alcoholism) at age 7, ever breastfed at age 7, cognitive ability at ages 7 and 11, conduct problems at ages 11 and 16, help for school backwardness at age 16, school attendance at age 16, drank alcohol at age 16, mathematics comprehension at age 16, voted in the 1979 general election at age 23, telephone in home at age 33, physical effort in job at age 33, voted in the 1987 general election at age 33, housing tenure at age 33, social capital score at age 33, participation in NCDS V at age 42, membership in organisations at age 42, and employer-provided pension at age 50.

To address unit non-response (cohort members not responding to the survey sweep of interest), we applied inverse probability weights provided with the cohort data. These weights are the inverse of each respondent's modelled probability of participating at the sweep, estimated from a wide range of characteristics measured earlier in the life course that predict drop-out. Respondents who resemble those lost to follow-up are up-weighted, so that the weighted sample more closely reflects the cohort as originally recruited. This reduces the risk that attrition related to SEP and health biases the associations of interest. Information on the amount of missing data is shown Tables A4-5 below.

**Table A4** The proportion of missing information in each variable in the SRH sample (N = 8,405)

| Variable | N missing | % missing |
| --- | --- | --- |
| Self-rated health | 42 | 0.50 |
| Adult social class | 2429 | 28.90 |
| Childhood social class | 827 | 9.84 |
| Child’s sex | 0 | 0 |
| Child’s ethnicity | 0 | 0 |
| Grandparental class | 1812 | 21.56 |
| Smoking | 1154 | 13.73 |
| Regular alcohol use | 1141 | 13.58 |
| Low exercise | 1165 | 13.86 |
| Malaise score | 1154 | 13.73 |
| Long-standing illness | 1150 | 13.68 |
| Marital status | 1320 | 15.70 |
| Number of children | 1709 | 20.33 |

**Table A5** The proportion of missing information in each variable in the biomarker sample (N = 6,309)

| Variable | N missing | % missing |
| --- | --- | --- |
| C-reactive protein (CRP) | 2295 | 36.38 |
| HDL cholesterol | 1323 | 36.38 |
| Total cholesterol | 1319 | 20.91 |
| Cholesterol ratio | 1325 | 21.00 |
| Glycated hemoglobin (HbA1c) | 1367 | 21.67 |
| NT-proBNP | 1423 | 22.56 |
| Childhood class | 604 | 9.57 |
| Adult class | 1581 | 25.06 |
| Child’s sex | 0 | 0 |
| Child’s ethnicity | 0 | 0 |
| Grandparental class | 1341 | 21.26 |
| Smoking | 691 | 10.95 |
| Regular alcohol use | 680 | 10.78 |
| Low exercise | 698 | 11.06 |
| Malaise score | 686 | 10.87 |
| Long-standing illness | 686 | 10.87 |
| Marital status | 818 | 12.97 |
| Number of children | 1126 | 17.85 |

A. **Mediation analyses in the full target population at the most recent sweep**

To assess the potential influence of selective attrition and outcome availability, we repeated the mediation analyses among all cohort members who were alive and had not emigrated by the most recent sweep (n=14,775), using multiple imputation to retain individuals with missing outcomes or other analysis variables. The substantive pattern of results was maintained in the full target population, indicating that the main conclusions were not materially affected by selective participation at the most recent sweep.

**Table A6** Interventional direct and indirect effects of childhood SEP on health, men (N= 7,420)

| Mediator | Direct effect | Indirect effect | Overall effect | rPM |
| --- | --- | --- | --- | --- |
| Self-rated health RR (CI) | | | | |
| Class | 0.82 (0.72, 0.97) | 0.90 (0.85, 0.94) | 0.74 (0.65, 0.87) | 0.33 (0.18, 0.91) |
| Housing | 0.78 (0.69, 0.91) | 0.95 (0.93, 0.98) | 0.74 (0.65, 0.87) | 0.14 (0.05, 0.41) |
| Education | 0.79 (0.71, 0.93) | 0.94 (0.90, 0.96) | 0.74 (0.65, 0.87) | 0.20 (0.11, 0.60) |
| Income | 0.75 (0.66, 0.88) | 0.99 (0.97, 1.00) | 0.74 (0.65, 0.87) | — |
| C-reactive protein RR (CI) | | | | |
| Class | 0.64 (0.51, 0.79) | 0.94 (0.89, 0.99) | 0.60 (0.49, 0.74) | 0.11 (0.01, 0.24) |
| Housing | 0.61 (0.49, 0.75) | 1.00 (0.97, 1.02) | 0.61 (0.49, 0.74) | — |
| Education | 0.64 (0.52, 0.80) | 0.94 (0.91, 0.99) | 0.60 (0.49, 0.74) | 0.10 (0.02, 0.25) |
| Income | 0.61 (0.49, 0.75) | 0.99 (0.98, 1.00) | 0.61 (0.49, 0.74) | — |
| Cholesterol ratio β (CI) | | | | |
| Class | -0.17 (-0.27, -0.04) | 0.01 (-0.04, 0.03) | -0.16 (-0.27, -0.05) | — |
| Housing | -0.15 (-0.26, -0.04) | -0.01 (-0.03, 0.00) | -0.16 (0.27, -0.05) | — |
| Education | -0.12 (-0.23, -0.02) | -0.04 (-0.05, 0.01) | -0.16 (-0.27, -0.05) | — |
| Income | -0.16 (-0.27, -0.05) | 0.00 (-0.01, 0.00) | -0.16 (-0.27, -0.05) | — |
| Glycated hemoglobin β (CI) | | | | |
| Class | -0.11 (-0.24, -0.04) | -0.04 (-0.06, 0.00) | -0.15 (-0.26, -0.06) | — |
| Housing | -0.13 (-0.25, -0.05) | -0.02 (-0.03, 0.00) | -0.15 (-0.26, -0.06) | — |
| Education | -0.13 (-0.25, -0.05) | -0.02 (-0.03, 0.01) | -0.15 (-0.26, -0.06) | — |
| Income | -0.14 (-0.25, -0.05) | -0.01 (-0.02, 0.00) | -0.15 (-0.26, -0.06) | — |
| NT-proBNP β (CI) | | | | |
| Class | 0.06 (-0.05, 0.16) | -0.01 (-0.05, 0.01) | 0.04 (-0.07, 0.14) | — |
| Housing | 0.04 (-0.07, 0.13) | 0.00 (-0.02, 0.01) | 0.04 ( -0.07, 0.14) | — |
| Education | 0.04 (-0.08, 0.14) | 0.00 (-0.02, 0.02) | 0.04 (-0.07, 0.14) | — |
| Income | 0.04 (-0.07, 0.14) | 0.00 (-0.01, 0.00) | 0.04 (-0.07, 0.14) | — |

**Table A7** Interventional direct and indirect effects of childhood SEP on health, women (N = 7,355)

| Mediator | Direct effect | Indirect effect | Overall effect | rPM |
| --- | --- | --- | --- | --- |
| Self-rated health RR (CI) | | | | |
| Class | 0.65 (0.58, 0.76) | 0.95 (0.93, 0.99) | 0.62 (0.55, 0.72) | 0.08 (0.02, 0.16) |
| Housing | 0.65 (0.58, 0.75) | 0.95 (0.92, 0.98) | 0.62 (0.55, 0.72) | 0.10 (0.04, 0.14) |
| Education | 0.65 (0.57, 0.76) | 0.95 (0.92, 0.97) | 0.62 (0.55, 0.72) | 0.09 (0.04, 0.18) |
| Income | 0.63 (0.56, 0.74) | 0.98 (0.97, 0.99) | 0.62 (0.55, 0.72) | 0.03 (0.01, 0.08) |
| C-reactive protein RR (CI) | | | | |
| Class | 0.66 (0.53, 0.80) | 1.01 (0.97, 1.03) | 0.67 (0.54, 0.79) | — |
| Housing | 0.69 (0.54, 0.81) | 0.96 (0.95, 0.99) | 0.67 (0.54, 0.79) | 0.08 (0.01, 0.16) |
| Education | 0.69 (0.56, 0.83) | 0.97 (0.93, 1.00) | 0.67 (0.54, 0.79) | — |
| Income | 0.66 (0.53, 0.79) | 1.01 (1.00, 1.03) | 0.67 (0.54, 0.79) | — |
| Cholesterol ratio β (CI) | | | | |
| Class | -0.30 (-0.37, -0.19) | -0.01 (-0.02, 0.01) | -0.31 (-0.38, -0.19) | — |
| Housing | -0.29 (-0.37, -0.19) | -0.01 (-0.03, 0.00) | -0.31 (-0.38, -0.19) | — |
| Education | -0.30 (-0.37, -0.20) | -0.01 (-0.03, 0.01) | -0.31 (-0.38, -0.19) | — |
| Income | -0.31 (-0.38, -0.19) | 0.00 (-0.01, 0.01) | -0.31 (-0.37, -0.19) | — |
| Glycated hemoglobin β (CI) | | | | |
| Class | -0.12 (-0.18, -0.05) | 0.00 (-0.01, 0.00) | -0.13 (-0.18, -0.06) | — |
| Housing | -0.12 (-0.17, -0.05) | -0.01 (-0.02, 0.00) | -0.13 (-0.18, -0.06) | — |
| Education | -0.12 (-0.18, -0.06) | 0.00 (-0.01, 0.01) | -0.12 (-0.18, -0.06) | — |
| Income | -0.12 (-0.18, -0.06) | 0.00 (-0.01, 0.00) | -0.13 (-0.18, -0.06) | — |
| NT-proBNP β (CI) | | | | |
| Class | 0.03 (-0.03, 0.11) | -0.01 (-0.02, 0.00) | 0.02 (-0.05, 0.10) | — |
| Housing | 0.02 (-0.04, 0.10) | 0.00 (-0.01, 0.01) | 0.02 (-0.05, 0.10) | — |
| Education | 0.03 (-0.04, 0.11) | -0.01 (-0.02, 0.01) | 0.02 (-0.05, 0.10) | — |
| Income | 0.02 (-0.05, 0.10) | 0.00 (0.00, 0.01) | 0.02 (-0.05, 0.10) | — |

B. **Alternative outcome specifications**

To assess whether our conclusions were sensitive to outcome operationalisation, we re-estimated the mediation models using alternative specifications for key biomarkers. CRP was modelled as a continuous outcome (mg/L) and values above 10 mg/L were set to missing to exclude likely acute infection. Cholesterol ratio and HbA1c were dichotomised to indicate elevated risk (cholesterol ratio >6) and diabetes (HbA1c ≥6.5%), respectively.

**Table A8** Interventional direct and indirect effects of childhood SEP on CRP (continuous)

| Panel A. Men | | | | |
| --- | --- | --- | --- | --- |
| Mediator | **Direct effect** | **Indirect effect** | **Overall effect** | **rPM** |
| Class | -0.35 (-0.53, -0.17) | -0.05 (-0.10, 0.00) | -0.40 (-0.56, -0.23) | 0.12 (-0.03, 0.31) |
| Housing | -0.32 (-0.49, -0.14) | -0.02 (-0.05, 0.01) | -0.33 (-0.50, -0.15) | — |
| Education | -0.28 (-0.44, -0.10) | -0.05 (-0.09, -0.01) | -0.33 (-0.50, -0.14) | 0.16 (0.04, 0.36) |
| Income | -0.33 (-0.50, -0.15) | 0.00 (-0.01, 0.01) | -0.33 (-0.50, 0.14) | — |
| Panel B. Women | | | | |
| Class | -0.66 (-0.87, -0.44) | 0.03 (0.00, 0.07) | -0.63 (-0.85, -0.40) | — |
| Housing | -0.55 (-0.74, -0.32) | -0.02 (-0.07, 0.00) | -0.57 (-0.76, -0.35) | 0.04 (0.00, 0.13) |
| Education | -0.54 (-0.73, -0.31) | -0.03 (-0.08, 0.02) | -0.57 (-0.76, -0.35) | — |
| Income | -0.58 (-0.77, -0.36) | 0.01 (-0.01, 0.03) | -0.57 (-0.76, -0.35) | — |

**Table A9** Interventional direct and indirect effects of childhood SEP on cholesterol ratio (binary)

| Panel A. Men | | | | |
| --- | --- | --- | --- | --- |
| Mediator | **Direct effect** | **Indirect effect** | **Overall effect** | **rPM** |
| Class | 0.94 (0.81, 1.06) | 0.99 (0.94, 1.03) | 0.93 (0.81, 1.03) | — |
| Housing | 0.92 (0.82, 1.05) | 1.00 (0.98, 1.01) | 0.92 (0.81, 1.05) | — |
| Education | 0.94 (0.82, 1.07) | 0.98 (0.95, 1.01) | 0.92 (0.81, 1.05) | — |
| Income | 0.92 (0.81, 1.04) | 1.00 (0.99, 1.00) | 0.92 (0.81, 1.04) | — |
| Panel B. Women | | | | |
| Class | 0.66 (0.54, 0.82) | 0.99 (0.95, 1.03) | 0.66 (0.53, 0.80) | — |
| Housing | 0.63 (0.52, 0.77) | 0.99 (0.96, 1.02) | 0.62 (0.52, 0.76) | — |
| Education | 0.63 (0.52, 0.76) | 0.99 (0.93, 1.03) | 0.62 (0.51, 0.76) | — |
| Income | 0.62 (0.52, 0.76) | 1.00 (0.98, 1.02) | 0.62 (0.51, 0.75) | — |

**Table A10** Interventional direct and indirect effects of childhood SEP on HbA1c (binary)

| Panel A. Men | | | | |
| --- | --- | --- | --- | --- |
| Mediator | **Direct effect** | **Indirect effect** | **Overall effect** | **rPM** |
| Class | 0.78 (0.59, 1.03) | 0.97 (0.89, 1.07) | 0.76 (0.59, 1.00) | — |
| Housing | 0.76 (0.58, 0.98) | 0.98 (0.92, 1.01) | 0.74 (0.57, 0.95) | — |
| Education | 0.77 (0.58, 0.97) | 0.96 (0.91, 1.03) | 0.74 (0.57, 0.95) | — |
| Income | 0.74 (0.57, 0.95) | 1.00 (0.98, 1.01) | 0.74 (0.57, 0.95) | — |
| Panel B. Women | | | | |
| Class | 0.71 (0.46, 1.11) | 0.94 (0.86, 1.01) | 0.67 (0.42, 1.04) | — |
| Housing | 0.70 (0.44, 0.95) | 0.94 (0.88, 1.00) | 0.66 (0.43, 0.89) | — |
| Education | 0.69 (0.44, 0.97) | 0.93 (0.87, 1.01) | 0.65 (0.43, 0.88) | — |
| Income | 0.66 (0.43, 0.89) | 1.00 (0.97, 1.03) | 0.66 0.43, 0.90) | — |

C. **Alternative medication assumptions**

To assess sensitivity to medication-related assumptions, we repeated the lipid analyses under alternative statin adjustment factors and conducted restriction-based analyses for NT-proBNP. Specifically, we assumed 20% and 30% reductions in total cholesterol among statin users (instead of 25%) (Table A11) and re-estimated NT-proBNP models excluding participants prescribed heart-failure–related medications (Table A12).

**Table A11** Interventional direct and indirect effects of childhood SEP on cholesterol ratio

| 20% reduction in total cholesterol | | | | |
| --- | --- | --- | --- | --- |
| Panel A. Men | | | | |
| Mediator | **Direct effect** | **Indirect effect** | **Overall effect** | **rPM** |
| Class | -0.18 (-0.35, 0.03) | -0.01 (-0.07, 0.05) | -0.19 (-0.35, -0.01) | — |
| Housing | -0.17 (-0.30, 0.01) | -0.02 (-0.05, 0.00) | -0.19 (-0.32, -0.01) | — |
| Education | -0.14 (-0.27, 0.03) | -0.05 (-0.09, -0.02) | -0.19 (-0.32, -0.01) | — |
| Income | -0.19 (-0.33, -0.01) | 0.00 (-0.01, 0.01) | -0.19 (-0.32, -0.01) | — |
| Panel B. Women | | | | |
| Class | -0.36 (-0.49, -0.21) | 0.00 (-0.03, 0.02) | -0.37 (-0.49, -0.22) | — |
| Housing | -0.38 (-0.49, -0.23) | -0.01 (-0.03, 0.00) | -0.39 (-0.49, -0.26) | — |
| Education | -0.39 (-0.51, -0.25) | 0.00 (-0.03, 0.03) | -0.39 (-0.50, -0.26) | — |
| Income | -0.39 (-0.50, -0.25) | 0.00 (-0.01, 0.01) | -0.39 (-0.49, -0.25) | — |
| 30% reduction in total cholesterol | | | | |
| Panel A. Men |  |  |  |  |
| Class | -0.21 (-0.42, 0.04) | -0.01 (-0.08, 0.05) | -0.22 (-0.41, -0.02) | — |
| Housing | -0.20 (-0.36, 0.00) | -0.02 (-0.05, 0.01) | -0.22 (-0.38, -0.01) | — |
| Education | -0.17 (-0.34, 0.03) | -0.05 (-0.10, -0.02) | -0.22 (-0.38, -0.01) | — |
| Income | -0.22 (-0.39, -0.01) | 0.00 (-0.01, 0.01) | -0.22 (-0.38, -0.01) | — |
| Panel B. Women |  |  |  |  |
| Class | -0.42 (-0.57, -0.26) | -0.01 (-0.03, 0.02) | -0.43 (-0.58, -0.26) | — |
| Housing | -0.45 (-0.57, -0.29) | -0.01 (-0.03, 0.00) | -0.46 (-0.58, -0.31) | — |
| Education | -0.46 (-0.59, -0.31) | 0.00 (-0.03, 0.03) | -0.46 (-0.59, -0.31) | — |
| Income | -0.46 (-0.58, -0.31) | 0.00 (-0.01, 0.01) | -0.46 (-0.58, -0.31) | — |

**Table A12** Interventional direct and indirect effects of childhood SEP on NT-proBNP

| Panel A. Men | | | | |
| --- | --- | --- | --- | --- |
| Mediator | **Direct effect** | **Indirect effect** | **Overall effect** | **rPM** |
| Class | 0.02 (-0.09, 0.12) | -0.01 (-0.05, 0.03) | 0.00 (-0.09, 0.12) | — |
| Housing | 0.03 (-0.06, 0.13) | 0.00 (-0.01, 0.02) | 0.04 (-0.06, 0.13) | — |
| Education | 0.04 (-0.07, 0.14) | 0.00 (-0.02, 0.03) | 0.04 (-0.06, 0.13) | — |
| Income | 0.04 (-0.06, 0.13) | 0.00 (-0.01, 0.01) | 0.04 (-0.06, 0.13) | — |
| Panel B. Women | | | | |
| Class | 0.06 (-0.01, 0.14) | -0.01 (-0.03, 0.01) | 0.05 (-0.02, 0.14) | — |
| Housing | 0.04 (-0.04, 0.11) | 0.00 (-0.01, 0.01) | 0.04 (-0.05, 0.11) | — |
| Education | 0.04 (-0.04, 0.11) | 0.00 (-0.02, 0.02) | 0.04 (-0.05, 0.11) | — |
| Income | 0.04 (-0.05, 0.11) | 0.00 (-0.01, 0.01) | 0.04 (-0.05, 0.11) | — |

D. **Sample restrictions**

To assess whether the findings were influenced by differences in outcome-collection timing or analytic-sample composition, we repeated the analyses using two restricted samples. First, biomarker analyses were limited to measurements collected during 2022–2023 (Tables A13-14), providing a more consistent collection period and reducing potential heterogeneity associated with measurements obtained at different stages of the most recent sweep. Second, self-rated health analyses were restricted to participants who also provided a blood sample (Table A15). This created a sample more comparable with that used for the biomarker analyses and assessed whether differences between the self-rated health and biomarker findings reflected outcome type rather than differences in sample composition.

**Table A13** Interventional direct and indirect effects of childhood SEP on HbA1c, restricted to measurements collected in 2022–2023

| Panel A. Men | | | | |
| --- | --- | --- | --- | --- |
| Mediator | **Direct effect** | **Indirect effect** | **Overall effect** | **rPM** |
| Class | -0.14 (-0.27, 0.02) | -0.02 (-0.06, 0.02) | -0.16 (-0.28, -0.03) | — |
| Housing | -0.14 (-0.26, -0.01) | -0.01 (-0.03, 0.01) | -0.15 (-0.27, -0.03) | — |
| Education | -0.14 (-0.26, -0.01) | -0.01 (-0.03, 0.01) | -0.15 (-0.27, -0.03) | — |
| Income | -0.15 (-0.27, -0.02) | 0.00 (-0.01, 0.01) | -0.15 (-0.27, -0.03) | — |
| Panel B. Women | | | | |
| Class | -0.13 (-0.21, -0.06) | -0.01 (-0.02, 0.00) | -0.13 (-0.21, -0.07) | — |
| Housing | -0.10 (-0.17, -0.03) | -0.01 (-0.01, 0.00) | -0.10 (-0.18, -0.04) | — |
| Education | -0.10 (-0.18, -0.03) | 0.00 (-0.02, 0.02) | -0.10 (-0.17, -0.04) | — |
| Income | -0.10 (-0.17, -0.04) | 0.00 (-0.01, 0.00) | -0.10 (-0.17, -0.04) | — |

Notes: Analytic sample sizes were 2,041 men and 2,075 women. Models using adult occupational class as the mediator excluded participants who were out of the labour force at age 55, reducing the samples to 1,827 men and 1,694 women.

**Table A14** Interventional direct and indirect effects of childhood SEP on cholesterol ratio, restricted to measurements collected in 2022–2023

| Panel A. Men | | | | |
| --- | --- | --- | --- | --- |
| Mediator | **Direct effect** | **Indirect effect** | **Overall effect** | **rPM** |
| Class | -0.16 (-0.33, -0.04) | 0.00 (-0.04, 0.06) | -0.15 (-0.31, -0.05) | — |
| Housing | -0.15 (-0.26, -0.02) | -0.01 (-0.04, 0.01) | -0.16 (-0.27, -0.04) | — |
| Education | -0.13 (-0.25, 0.01) | -0.03 (-0.06, -0.01) | -0.16 (-0.27, -0.03) | — |
| Income | -0.16 (-0.27, -0.03) | 0.00 (-0.01, 0.01) | -0.16 (-0.27, -0.03) | — |
| Panel B. Women | | | | |
| Class | -0.31 (-0.43, -0.19) | 0.00 (-0.03, 0.02) | -0.32 (-0.43, -0.19) | — |
| Housing | -0.30 (-0.41, -0.18) | -0.01 (-0.03, 0.00) | -0.31 (-0.41, -0.19) | — |
| Education | -0.32 (-0.43, -0.20) | 0.01 (-0.02, 0.04) | -0.31 (-0.42, -0.19) | — |
| Income | -0.31 (-0.42, -0.19) | 0.00 (-0.01, 0.01) | -0.31 (-0.42, -0.19) | — |

Notes: Analytic sample sizes were 2,055 men and 2,095 women. Models using adult occupational class as the mediator excluded participants who were out of the labour force at age 55, reducing the samples to 1,840 men and 1,710 women.

**Table A15** Interventional direct and indirect effects of childhood SEP on SRH, restricted to blood-sample providers

| Panel A. Men | | | | |
| --- | --- | --- | --- | --- |
| Mediator | **Direct effect** | **Indirect effect** | **Overall effect** | **rPM** |
| Class | 0.92 (0.78, 1.09) | 0.87 (0.82, 0.93) | 0.81 (0.69, 0.95) | 0.63 (0.22, 3.73) |
| Housing | 0.81 (0.68, 0.94) | 0.96 (0.92, 1.00) | 0.77 (0.65, 0.92) | 0.16 (-0.01, 0.41) |
| Education | 0.83 (0.69, 0.98) | 0.93 (0.89, 0.97) | 0.77 (0.65, 0.92) | 0.27 (0.09, 0.83) |
| Income | 0.78 (0.66, 0.93) | 0.99 (0.97, 1.00) | 0.77 (0.65, 0.92) | 0.04 (-0.01, 0.15) |
| Panel B. Women | | | | |
| Class | 0.67 (0.52, 0.81) | 0.95 (0.90, 0.99) | 0.63 (0.50, 0.78) | 0.09 (0.02, 0.26) |
| Housing | 0.65 (0.55, 0.77) | 0.95 (0.92, 0.99) | 0.62 (0.52, 0.74) | 0.08 (0.02, 0.17) |
| Education | 0.65 (0.54, 0.77) | 0.96 (0.92, 1.00) | 0.62 (0.52, 0.74) | 0.08 (0.01, 0.18) |
| Income | 0.63 (0.53, 0.75) | 0.98 (0.95, 1.00) | 0.62 (0.52, 0.74) | 0.03 (0.00, 0.09) |

Notes: Analytic sample sizes were 3,146 men and 3,160 women. Models using adult occupational class as the mediator excluded participants who were out of the labour force at age 55, reducing the analytic samples to 2,791 men and 2,550 women.

E. **Income mediation robustness checks**

To assess whether the estimated mediation through income depended on how income was defined or when it was measured, we repeated the income analyses using alternative operationalisations and measurement time points. The first set of analyses examined whether the findings were sensitive to alternative ways of categorising or scaling income (Tables A16-17). The second examined whether the results were robust to measuring income at different stages of adulthood (Tables A18-19), rather than being specific to the timing of the income measure used in the primary analysis.

**Table A16** Interventional direct and indirect effects of childhood SEP on health, men, using alternative income operationalisations

| Men | Direct effect | Indirect effect | Overall effect | rPM |
| --- | --- | --- | --- | --- |
| Self-rated health RR (CI) | | | | |
| Log-income age 55 | 0.78 (0.68, 0.88) | 0.99 (0.97, 1.00) | 0.77 (0.67, 0.88) | 0.04 (0.00, 0.11) |
| high = top 40% | 0.79 (0.69, 0.90) | 0.97 (0.95, 0.99) | 0.77 (0.66, 0.87) | 0.09 (0.04, 0.23) |
| high = top 80% | 0.77 (0.67, 0.88) | 1.00 (0.98, 1.01) | 0.77 (0.66, 0.87) | — |
| high = ≥ 60% of median | 0.77 (0.68, 0.89) | 0.99 (0.98, 1.01) | 0.77 (0.66, 0.87) | — |
| C-reactive protein RR (CI) | | | | |
| Log-income age 55 | 0.62 (0.49, 0.85) | 1.00 (0.99, 1.01) | 0.62 (0.49, 0.84) | — |
| high = top 40% | 0.64 (0.50, 0.82) | 0.98 (0.95, 1.01) | 0.63 (0.48, 0.80) | — |
| high = top 80% | 0.63 (0.48, 0.80) | 1.00 (0.99, 1.04) | 0.63 (0.48, 0.80) | — |
| high = ≥ 60% of median | 0.63 (0.49, 0.81) | 1.00 (0.98, 1.03) | 0.63 (0.48, 0.80) | — |
| Cholesterol ratio β (CI) | | | | |
| Log-income age 55 | -0.14 (-0.28, 0.01) | 0.00 (-0.01, 0.01) | -0.14 (-0.28, 0.01) | — |
| high = top 40% | -0.13 (-0.26, 0.00) | -0.01 (-0.02, 0.00) | -0.14 (-0.27, 0.00) | — |
| high = top 80% | -0.14 (-0.26, 0.00) | 0.00 (-0.01, 0.02) | -0.14 (-0.27, 0.00) | — |
| high = ≥ 60% of median | -0.14 (-0.26, 0.00) | 0.00 (-0.01, 0.01) | -0.14 (-0.27, 0.00) | — |
| Glycated hemoglobin β (CI) | | | | |
| Log-income age 55 | -0.14 (-0.24, -0.04) | 0.00 (-0.01, 0.01) | -0.14 (-0.24, -0.03) | — |
| high = top 40% | -0.14 (-0.22, -0.04) | 0.00 (-0.01, 0.01) | -0.14 (-0.22, -0.04) | — |
| high = top 80% | -0.14 (-0.22, -0.04) | 0.00 (-0.01, 0.01) | -0.14 (-0.22, -0.04) | — |
| high = ≥ 60% of median | -0.14 (-0.22, -0.04) | 0.00 (-0.01, 0.01) | -0.14 (-0.22, -0.04) | — |
| NT-proBNP β CI) | | | | |
| Log-income age 55 | 0.04 (-0.03, 0.14) | 0.00 (-0.01, 0.00) | 0.04 (-0.03, 0.14) | — |
| high = top 40% | 0.05 (-0.03, 0.15) | 0.00 (-0.01, 0.00) | 0.04 (-0.03, 0.14) | — |
| high = top 80% | 0.04 (-0.03, 0.14) | 0.00 (0.00, 0.00) | 0.04 (-0.03, 0.14) | — |
| high = ≥ 60% of median | 0.04 (-0.03, 0.14) | 0.00 (0.00, 0.00) | 0.04 (-0.03, 0.14) | — |

**Table A17** Interventional direct and indirect effects of childhood SEP on health, women, using alternative income operationalisations

| Women | Direct effect | Indirect effect | Overall effect | rPM |
| --- | --- | --- | --- | --- |
| Self-rated health RR (CI) | | | | |
| Log-income age 55 | 0.66 (0.57, 0.76) | 0.98 (0.96, 1.00) | 0.65 (0.56, 0.75) | 0.03 (0.01, 0.08) |
| high = top 40% | 0.65 (0.57, 0.76) | 0.99 (0.98, 1.01) | 0.64 (0.56, 0.75) | — |
| high = top 80% | 0.65 (0.56, 0.76) | 0.99 (0.97, 1.00) | 0.64 (0.56, 0.75) | — |
| high = ≥ 60% of median | 0.65 (0.56, 0.76) | 0.99 (0.98, 1.00) | 0.64 (0.56, 0.75) | — |
| C-reactive protein RR (CI) | | | | |
| Log-income age 55 | 0.66 (0.54, 0.78) | 1.02 (0.99, 1.05) | 0.67 (0.56, 0.79) | — |
| high = top 40% | 0.66 (0.53, 0.78) | 1.01 (0.98, 1.04) | 0.66 (0.54, 0.78) | — |
| high = top 80% | 0.66 (0.53, 0.78) | 1.00 (0.99, 1.01) | 0.66 (0.53, 0.79) | — |
| high = ≥ 60% of median | 0.66 (0.53, 0.78) | 1.00 (0.99, 1.01) | 0.66 (0.53, 0.79) | — |
| Cholesterol ratio β (CI) | | | | |
| Log-income age 55 | -0.29 (-0.39, -0.17) | 0.00 (-0.01, 0.01) | -0.29 (-0.39, -0.16) | — |
| high = top 40% | -0.28 (-0.39, -0.19) | 0.00 (-0.01, 0.01) | -0.28 (-0.39, -0.19) | — |
| high = top 80% | -0.28 (-0.39, -0.19) | 0.00 (0.00, 0.01) | -0.28 (-0.39, -0.19) | — |
| high = ≥ 60% of median | -0.28 (-0.38, -0.18) | 0.00 (-0.01, 0.00) | -0.28 (-0.39, -0.19) | — |
| Glycated hemoglobin β (CI) | | | | |
| Log-income age 55 | -0.13 (-0.21, -0.07) | 0.00 (-0.01, 0.00) | -0.13 (-0.21, -0.07) | — |
| high = top 40% | -0.14 (-0.20, -0.07) | 0.00 (-0.01, 0.00) | -0.14 (-0.20, -0.08) | — |
| high = top 80% | -0.14 (-0.20, -0.07) | 0.00 (0.00, 0.00) | -0.14 (-0.20, -0.07) | — |
| high = ≥ 60% of median | -0.14 (-0.20, -0.07) | 0.00 (0.00, 0.00) | -0.14 (-0.20, -0.07) | — |
| NT-proBNP β (CI) | | | | |
| Log-income age 55 | 0.02 (-0.06, 0.09) | 0.00 (-0.01, 0.01) | 0.02 (-0.05, 0.08) | — |
| high = top 40% | 0.02 (-0.05, 0.10) | 0.00 (-0.01, 0.01) | 0.02 (-0.05, 0.10) | — |
| high = top 80% | 0.02 (-0.05, 0.10) | 0.00 (-0.01, 0.00) | 0.02 (-0.05, 0.10) | — |
| high = ≥ 60% of median | 0.02 (-0.05, 0.10) | 0.00 (0.00, 0.00) | 0.02 (-0.05, 0.10) | — |

**Table A18** Interventional direct and indirect effects of childhood SEP on health, men, using **income measured at alternative time points** at age 50

|  | Direct effect | Indirect effect | Overall effect | rPM |
| --- | --- | --- | --- | --- |
| Self-rated health RR (CI) | | | | |
| Log-income age 50 | 0.76 (0.68, 0.83) | 0.98 (0.95, 0.99) | 0.74 (0.66, 0.82) | 0.07 (0.03, 0.18) |
| high = top 40% | 0.76 (0.68, 0.84) | 0.98 (0.94, 0.99) | 0.75 (0.66, 0.82) | 0.07 (0.03, 0.21) |
| C-reactive protein RR (CI) | | | | |
| Log-income age 50 | 0.65 (0.51, 0.83) | 0.98 (0.94, 1.01) | 0.64 (0.49, 0.82) | — |
| high = top 40% | 0.64 (0.49, 0.82) | 1.00 (0.97, 1.03) | 0.64 (0.49, 0.82) | — |
| Cholesterol ratio β (CI) | | | | |
| Log-income age 50 | -0.13 (-0.27, 0.01) | -0.01 (-0.02, 0.00) | -0.14 (-0.28, 0.00) | — |
| high = top 40% | -0.13 (-0.26, 0.01) | -0.01 (-0.03, 0.00) | -0.14 (-0.28, 0.00) | — |
| Glycated hemoglobin β (CI) | | | | |
| Log-income age 50 | -0.13 (-0.24, -0.03) | -0.01 (-0.02, 0.01) | -0.14 (-0.24, -0.04) | — |
| high = top 40% | -0.13 (-0.23, -0.03) | -0.01 (-0.02, 0.00) | -0.14 (-0.24, -0.04) | — |
| NT-proBNP β (CI) | | | | |
| Log-income age 50 | 0.04 (-0.04, 0.1) | 0.00 (-0.01, 0.02) | 0.04 (-0.04, 0.14) | — |
| high = top 40% | 0.04 (-0.04, 0.1) | 0.01 (-0.01, 0.02) | 0.04 (-0.04, 0.14) | — |

**Table A19** Interventional direct and indirect effects of childhood SEP on health, women, using **income measured at alternative time points** at age 50

|  | Direct effect | Indirect effect | Overall effect | rPM |
| --- | --- | --- | --- | --- |
| Self-rated health RR (CI) | | | | |
| Log-income age 50 | 0.65 (0.56, 0.75) | 1.00 (0.99, 1.00) | 0.65 (0.56, 0.74) | — |
| high = top 40% | 0.65 (0.56, 0.75) | 0.99 (0.98, 1.00) | 0.65 (0.56, 0.74) | — |
| C-reactive protein RR (CI) | | | | |
| Log-income age 50 | 0.63 (0.54, 0.73) | 1.00 (0.99, 1.01) | 0.63 (0.54, 0.72) | — |
| high = top 40% | 0.66 (0.54, 0.81) | 1.00 (0.98, 1.01) | 0.66 (0.54, 0.81) | — |
| Cholesterol ratio β (CI) | | | | |
| Log-income age 50 | -0.29 (-0.38, -0.18) | 0.00 (-0.01, 0.00) | -0.29 (-0.39, -0.18) | — |
| high = top 40% | -0.29 (-0.39, -0.19) | 0.00 (-0.01, 0.00) | -0.29 (-0.39, -0.18) | — |
| Glycated hemoglobin β (CI) | | | | |
| Log-income age 50 | -0.13 (-0.21, -0.07) | 0.00 (-0.01, 0.00) | -0.14 (-0.21, -0.07) | — |
| high = top 40% | -0.13 (-0.21, -0.07) | 0.00 (-0.01, 0.00) | -0.14 (-0.21, -0.07) | — |
| NT-proBNP β (CI) | | | | |
| Log-income age 50 | 0.02 (-0.05, 0.08) | 0.00 (0.00, 0.00) | 0.02 (-0.05, 0.09) | — |
| high = top 40% | 0.02 (-0.05, 0.09) | 0.00 ( -0.01, 0.00) | 0.02 (-0.05, 0.09) | — |

F. **Alternative indicators of childhood SEP**

To assess whether the findings were specific to the primary definition of childhood SEP based on paternal occupational class at birth, we repeated the mediation analyses using two alternative indicators capturing different dimensions of childhood socioeconomic circumstances: housing tenure at age 7 and parental education (Appendix Tables A20–23). Housing tenure at age 7 was classified as owner-occupied or rented. Participants whose family home was recorded as owner-occupied were classified as owners, while those living in privately rented, council-rented, or other rented accommodation were classified as renters. Parental education was derived from the reported ages at which the cohort member’s father and mother left full-time education. The highest observed school-leaving age across the two parents was used. Parental education was then classified as neither parent remained in full-time education until age 16 versus at least one parent remained in full-time education until age 16 or older. Because the parents of cohort members born in 1958 would generally have completed schooling before the compulsory school-leaving age was raised to 16, remaining in education until at least age 16 provides a conservative indicator of post-compulsory parental education.

The sensitivity analyses produced findings that were substantively consistent with the main analysis, suggesting that our main conclusions were not dependent on the specific primary indicator of childhood SEP. However, because housing tenure was measured at age 7, factors arising between birth and age 7 may additionally confound the associations of age-7 housing tenure with adult SEP and later health. The housing-tenure sensitivity analysis should therefore be interpreted primarily as evidence of robustness to an alternative childhood SEP indicator, rather than as the isolated causal effect of housing tenure at age 7.

**Table A20** Interventional direct and indirect effects of housing tenure on health, men

| Mediator | Direct effect | Indirect effect | Overall effect | rPM |
| --- | --- | --- | --- | --- |
| Self-rated health RR (CI) | | | | |
| Class | 0.84 (0.73, 0.94) | 0.92 (0.89, 0.96) | 0.77 (0.68, 0.87) | 0.29 (0.14, 0.55) |
| Housing | 0.80 (0.71, 0.90) | 0.95 (0.93, 0.98) | 0.76 (0.68, 0.86) | 0.17 (0.07, 0.29) |
| Education | 0.80 (0.71, 0.90) | 0.95 (0.93, 0.97) | 0.76 (0.68, 0.86) | 0.18 (0.07, 0.37) |
| Income | 0.78 (0.70, 0.89) | 0.98 (0.96, 0.99) | 0.76 (0.68, 0.86) | 0.08 (0.04, 0.22) |
| C-reactive protein RR (CI) | | | | |
| Class | 0.81(0.71, 0.99) | 0.96 (0.91, 0.98) | 0.77 (0.67, 0.95) | 0.17 (0.06, 1.22) |
| Housing | 0.82 (0.68, 0.98) | 1.00 (0.97, 1.03) | 0.82 (0.68, 0.98) | — |
| Education | 0.85 (0.72, 1.00) | 0.95 (0.92, 0.99) | 0.81 (0.67, 0.97) | — |
| Income | 0.82 (0.68, 0.97) | 0.99 (0.98, 1.01) | 0.82 (0.67, 0.97) | — |
| Cholesterol ratio β (CI) | | | | |
| Class | -0.17 (-0.31, -0.03) | 0.01 (-0.01, 0.04) | -0.17 (-0.29, -0.02) | — |
| Housing | -0.16 (-0.28, -0.04) | -0.01 (-0.04, 0.01) | -0.17 (-0.30, -0.05) | — |
| Education | -0.16 (-0.29, -0.04) | -0.01 (-0.03, 0.01) | -0.17 (-0.30, -0.05) | — |
| Income | -0.17 (-0.29, -0.05) | 0.00 (-0.01, 0.00) | -0.17 (-0.30, -0.05) | — |
| Glycated hemoglobin β (CI) | | | | |
| Class | -0.11 (-0.20, -0.02) | -0.02 (-0.04, 0.00) | -0.13 (-0.21, -0.03) | — |
| Housing | -0.13 (-0.24, 0.00) | -0.01 (-0.03, 0.00) | -0.14 (-0.25, -0.02) | — |
| Education | -0.14 (-0.24, -0.01) | 0.00 (-0.02, 0.01) | -0.14 (-0.25, -0.02) | — |
| Income | -0.14 (-0.24, -0.02) | 0.00 (-0.01, 0.00) | -0.15 (-0.25, -0.02) | — |
| NT-proBNP β (CI) | | | | |
| Class | 0.03 (-0.04, 0.13) | -0.01 (-0.03, 0.00) | 0.02 (-0.04, 0.11) | — |
| Housing | 0.00 (-0.07, 0.09) | 0.00 (-0.01, 0.01) | 0.00 (-0.08, 0.09) | — |
| Education | 0.00 (-0.08, 0.09) | 0.00 (-0.01, 0.02) | 0.00 (-0.08, 0.09) | — |
| Income | 0.00 (-0.07, 0.09) | 0.00 (-0.01, 0.01) | 0.00 (-0.08, 0.09) | — |

**Table A21** Interventional direct and indirect effects of housing tenure on health, women

| Mediator | Direct effect | Indirect effect | Overall effect | rPM |
| --- | --- | --- | --- | --- |
| Self-rated health RR (CI) | | | | |
| Class | 0.79 (0.69, 0.91) | 0.95 (0.91, 1.00) | 0.75 (0.66, 0.86) | — |
| Housing | 0.72 (0.64, 0.83) | 0.95 (0.93, 0.98) | 0.69 (0.61, 0.78) | 0.10 (0.06, 0.20) |
| Education | 0.72 (0.65, 0.83) | 0.95 (0.92, 0.97) | 0.68 (0.61, 0.78) | 0.12 (0.06, 0.22) |
| Income | 0.69 (0.62, 0.78) | 1.00 (0.98, 1.00) | 0.68 (0.61, 0.78) | — |
| C-reactive protein RR (CI) | | | | |
| Class | 0.65 (0.56, 0.80) | 0.99 (0.96, 1.04) | 0.65 (0.55, 0.79) | — |
| Housing | 0.71 (0.61, 0.84) | 0.96 (0.90, 1.00) | 0.68 (0.59, 0.79) | — |
| Education | 0.72 (0.62, 0.85) | 0.95 (0.91, 1.00) | 0.68 (0.60, 0.78) | — |
| Income | 0.68 (0.59, 0.78) | 1.01 (1.00, 1.03) | 0.68 (0.60, 0.79) | — |
| Cholesterol ratio β (CI) | | | | |
| Class | -0.24 (-0.34, -0.16) | 0.00 (-0.02, 0.01) | -0.25 (-0.34, -0.16) | — |
| Housing | -0.27 (-0.35, -0.18) | -0.01 (-0.03, 0.00) | -0.29 (-0.37, -0.19) | — |
| Education | -0.28 (-0.37, -0.18) | 0.00 (-0.02, 0.02) | -0.29 (-0.37, -0.19) | — |
| Income | -0.29 (-0.37, -0.19) | 0.00 (0.00, 0.01) | -0.29 (-0.37, -0.19) | — |
| Glycated hemoglobin β (CI) | | | | |
| Class | -0.08 (-0.17, 0.00) | 0.00 (-0.01, 0.01) | -0.08 ( -0.17, 0.00) | — |
| Housing | -0.09 (-0.16, -0.01) | 0.00 (-0.02, 0.00) | -0.09 (-0.17, -0.02) | — |
| Education | -0.09 (-0.17, -0.01) | -0.01 (-0.02, 0.01) | -0.09 (-0.17, -0.02) | — |
| Income | -0.09 (-0.17, -0.02) | 0.00 (0.00, 0.00) | -0.09 (-0.17, -0.02) | — |
| NT-proBNP β (CI) | | | | |
| Class | -0.02 (-0.09, 0.04) | 0.00 (-0.0, 0.01) | -0.03 (-0.10, 0.04) | — |
| Housing | -0.04 (-0.10, 0.03) | 0.01 (0.00, 0.02) | -0.03 (-0.09, 0.03) | — |
| Education | -0.02 (-0.07, 0.05) | -0.01 (-0.03, 0.00) | -0.03 (-0.09, 0.03) | — |
| Income | -0.03 (-0.09, 0.03) | 0.00 (0.00, 0.01) | -0.03 (-0.09, 0.03) | — |

**Table A22** Interventional direct and indirect effects of parental education on health, men

| Mediator | Direct effect | Indirect effect | Overall effect | rPM |
| --- | --- | --- | --- | --- |
| Self-rated health RR (CI) | | | | |
| Class | 0.77 (0.64, 0.90) | 0.89 (0.86, 0.95) | 0.69 (0.59, 0.82) | 0.27 (0.11, 0.49) |
| Housing | 0.70 (0.62, 0.78) | 0.96 (0.93, 0.99) | 0.68 (0.60, 0.75) | 0.09 (0.03, 0.17) |
| Education | 0.73 (0.65, 0.81) | 0.92 (0.88, 0.96) | 0.67 (0.59, 0.74) | 0.19 (0.08, 0.32) |
| Income | 0.68 (0.61, 0.76) | 0.99 (0.97, 1.00) | 0.67 (0.60, 0.75) | — |
| C-reactive protein RR (CI) | | | | |
| Class | 0.79 (0.61, 1.01) | 0.90 (0.81, 0.95) | 0.71 (0.53, 0.91) | — |
| Housing | 0.73 (0.52, 0.91) | 1.00 (0.98, 1.02) | 0.73 (0.52, 0.91) | — |
| Education | 0.79 (0.58, 1.02) | 0.91 (0.85, 0.98) | 0.72 (0.52, 0.90) | — |
| Income | 0.73 (0.52, 0.91) | 1.00 (0.99, 1.00) | 0.73 (0.52, 0.91) | — |
| Cholesterol ratio β (CI) | | | | |
| Class | -0.08 (-0.20, 0.06) | 0.00 (-0.04, 0.05) | -0.08 (-0.21, 0.06) | — |
| Housing | -0.08 (-0.23, 0.03) | -0.01 (-0.04, 0.00) | -0.09 (-0.24, 0.02) | — |
| Education | -0.03 (-0.20, 0.08) | -0.05 (-0.09, -0.01) | -0.09 (-0.24, 0.03) | — |
| Income | -0.09 (-0.24, 0.03) | 0.00 (-0.01, 0.01) | -0.09 (-0.24, 0.02) | — |
| Glycated hemoglobin β (CI) | | | | |
| Class | -0.07 (-0.18, 0.07) | -0.02 (-0.05, 0.01) | -0.10 (-0.20, 0.03) | — |
| Housing | -0.11 (-0.23, -0.02) | 0.00 (-0.02, 0.01) | -0.12 (-0.24, -0.03) | — |
| Education | -0.11 (-0.24, -0.01) | -0.01 (-0.04, 0.02) | -0.12 (-0.24, -0.02) | — |
| Income | -0.12 (-0.24, -0.02) | 0.00 (-0.01, 0.00) | -0.12 (-0.24, -0.03) | — |
| NT-proBNP β (CI) | | | | |
| Class | -0.01 (-0.14, 0.10) | -0.02 (-0.05, 0.02) | -0.03 (-0.13, 0.06) | — |
| Housing | -0.02 (-0.10, 0.07) | 0.00 (-0.01, 0.01) | -0.02 (-0.10, 0.07) | — |
| Education | -0.02 (-0.10, 0.06) | 0.01 (-0.01, 0.03) | -0.02 (-0.10, 0.07) | — |
| Income | -0.02 (-0.10, 0.08) | 0.00 (0.00, 0.00) | -0.02 (-0.10, 0.07) | — |

**Table A23** Interventional direct and indirect effects of parental education on health, women

| Mediator | Direct effect | Indirect effect | Overall effect | rPM |
| --- | --- | --- | --- | --- |
| Self-rated health RR (CI) | | | | |
| Class | 0.77 (0.66, 0.92) | 0.95 (0.91, 1.00) | 0.73 (0.63, 0.90) | — |
| Housing | 0.71 (0.60, 0.80) | 0.94 (0.90, 0.98) | 0.67 (0.56, 0.77) | 0.12 (0.04, 0.21) |
| Education | 0.71 (0.61, 0.82) | 0.93 (0.89, 0.97) | 0.66 (0.56, 0.76) | 0.15 (0.05, 0.26) |
| Income | 0.68 (0.57, 0.77) | 0.99 (0.97, 1.00) | 0.67 (0.57, 0.76) | — |
| C-reactive protein RR (CI) | | | | |
| Class | 0.70 (0.59, 0.86) | 0.99 (0.93, 1.06) | 0.70 (0.57, 0.86) | — |
| Housing | 0.74 (0.59, 0.93) | 0.95 (0.91, 0.99) | 0.71 (0.58, 0.87) | 0.12 (0.01, 0.57) |
| Education | 0.76 (0.62, 0.94) | 0.93 (0.87, 1.00) | 0.71 (0.58, 0.87) | — |
| Income | 0.70 (0.56, 0.87) | 1.01 (1.00, 1.05) | 0.71 (0.58, 0.87) | — |
| Cholesterol ratio β (CI) | | | | |
| Class | -0.18 (-0.31, -0.06) | -0.01 (-0.05, 0.01) | -0.19 (-0.32, -0.08) | — |
| Housing | -0.13 (-0.27, -0.01) | -0.02 (-0.04, 0.00) | -0.16 (-0.28, -0.05) | — |
| Education | -0.13 (-0.26, -0.01) | -0.03 (-0.06, -0.01) | -0.16 (-0.28, -0.04) | — |
| Income | -0.16 (-0.28, -0.04) | 0.00 (-0.01, 0.01) | -0.16 (-0.28, -0.05) | — |
| Glycated hemoglobin β (CI) | | | | |
| Class | -0.10 (-0.20, -0.01) | 0.00 (-0.02, 0.01) | -0.11 (-0.19, 0.00) | — |
| Housing | -0.06 (-0.14, 0.03) | -0.01 (-0.03, 0.00) | -0.07 (-0.14, 0.02) | — |
| Education | -0.06 (-0.14, 0.03) | -0.01 (-0.02, 0.01) | -0.07 (-0.14, 0.02) | — |
| Income | -0.07 (-0.15, 0.02) | 0.00 (-0.01, 0.00) | -0.07 (-0.14, 0.02) | — |
| NT-proBNP β (CI) | | | | |
| Class | 0.00 (-0.09, 0.07) | 0.00 (-0.02, 0.02) | 0.00 (-0.10, 0.07) | — |
| Housing | 0.00 (-0.08, 0.07) | 0.01 (-0.01, 0.02) | 0.01 (-0.07, 0.07) | — |
| Education | 0.01 (-0.06, 0.09) | -0.01 (-0.03, 0.01) | 0.01 (-0.07, 0.07) | — |
| Income | 0.00 (-0.07, 0.07) | 0.00 (0.00, 0.01) | 0.01 (-0.07, 0.07) | — |

**Reference**

**Choi J, Dekkers OM, le Cessie S. How measurements affected by medication use are reported and handled in observational research: a literature review. Pharmacoepidemiol Drug Saf 2022; 31: 739–48. https://doi.org/10.1002/pds.5437**

**Eastwood SV, Mathur R, Atkinson M, et al. Algorithms for the capture and adjudication of prevalent and incident diabetes in UK Biobank. PLoS One 2016; 11: e0162388. https://doi.org/10.1371/journal.pone.0162388**

**Edwards JE, Moore RA. Statins in hypercholesterolaemia: a dose-specific meta-analysis of lipid changes in randomised, double blind trials. BMC Fam Pract 2003; 4: 18. https://doi.org/10.1186/1471-2296-4-18**

**Januzzi JL Jr, Chen-Tournoux AA, Christenson RH, et al. N-terminal pro-B-type natriuretic peptide in the emergency department: the ICON-RELOADED study. J Am Coll Cardiol 2018; 71: 1191–200. https://doi.org/10.1016/j.jacc.2018.01.021**

**Law MR, Wald NJ, Rudnicka AR. Quantifying effect of statins on low density lipoprotein cholesterol, ischaemic heart disease, and stroke: systematic review and meta-analysis. BMJ 2003; 326: 1423. https://doi.org/10.1136/bmj.326.7404.1423**

**Mostafa T, Narayanan M, Pongiglione B, et al. Improving the plausibility of the missing at random assumption in the 1958 British birth cohort: a pragmatic data driven approach. CLS working paper 2020/6. London: UCL Centre for Longitudinal Studies, 2020.**

**Ponikowski P, Voors AA, Anker SD, et al. 2016 ESC Guidelines for the diagnosis and treatment of acute and chronic heart failure: the Task Force for the diagnosis and treatment of acute and chronic heart failure of the European Society of Cardiology (ESC). Developed with the special contribution of the Heart Failure Association (HFA) of the ESC. Eur Heart J 2016; 37: 2129–200. https://doi.org/10.1093/eurheartj/ehw128**

**Tobin MD, Sheehan NA, Scurrah KJ, Burton PR. Adjusting for treatment effects in studies of quantitative traits: antihypertensive therapy and systolic blood pressure. Stat Med 2005; 24: 2911–35. https://doi.org/10.1002/sim.2165**

White IR, Royston P, Wood AM. Multiple imputation using chained equations: issues and guidance for practice. Stat Med 2011; 30: 377–99. https://doi.org/10.1002/sim.4067

**Yancy CW, Jessup M, Bozkurt B, et al. 2017 ACC/AHA/HFSA focused update of the 2013 ACCF/AHA guideline for the management of heart failure: a report of the American College of Cardiology/American Heart Association Task Force on Clinical Practice Guidelines and the Heart Failure Society of America. Circulation 2017; 136: e137–61.** <https://doi.org/10.1161/CIR.0000000000000509>
